# The efficacy and safety of pembrolizumab in the treatment of HER2-negative advanced gastric or gastroesophageal junction cancer: a systematic review and meta-analysis of emerging clinical data

**DOI:** 10.3389/fimmu.2026.1862751

**Published:** 2026-08-19

**Authors:** Ying Yang, Lingli Jiang, Genming Zhang, Ke Li, Daorui Li

**Affiliations:** 1 Oncology Department, Guang’anmen Hospital of China Academy of Chinese Medical Sciences, Beijing, China; 2 Graduate School, Beijing University of Chinese Medicine, Beijing, China

**Keywords:** advanced gastric cancer, advanced gastroesophageal junction cancer, meta-analysis, pembrolizumab, systematic review

## Abstract

**Introduction:**

Pembrolizumab, an immune checkpoint inhibitor (ICI) targeting programmed cell death protein 1 (PD-1), has demonstrated significant clinical value in the treatment of human epidermal growth factor receptor 2 (HER2)-negative advanced gastric cancer (GC) and gastroesophageal junction cancer (GEJC). However, due to the relatively short duration of existing studies, inconsistent survival benefits among patients, and the lack of a standardized risk assessment system, its efficacy and safety still remain controversial.

**Materials and methods:**

By systematically querying the following English-language databases, including PubMed, Embase, Web of Science, Scopus, Ovid, Cochrane Library, and CINAHL, along with the clinical trial registry ClinicalTrials.gov, studies reporting clinical efficacy and safety outcomes of pembrolizumab in patients with HER2-negative advanced GC or GEJC were identified. A meta analysis was subsequently conducted to pool objective response rate (ORR), duration of response (DoR), overall survival (OS), progression free survival (PFS), 12/24-month overall survival rate (OS-12/24), 12/24-month progression-free survival rate (PFS-12/24), as well as treatment related adverse events (TRAEs) and immune related adverse events (irAEs). Comprehensive subgroup analyses were further performed based on PD-L1 combined positive score (CPS), age, region, chemo backbone, and median follow-up.

**Results:**

Nine studies encompassing 3406 patients with HER2-negative advanced GC or GEJC were included. In randomized controlled trials (RCTs), pembrolizumab plus chemotherapy significantly improved ORR (OR 1.57, 95%CI:1.35-1.81) and OS (HR 0.82, 95%CI:0.75-0.89) compared with chemotherapy alone. Pooled median PFS from RCTs in the pembrolizumab arm was 6.77 months (95%CI:6.19-7.36) and from single-arm studies was 6.53 months (95%CI:4.59-9.31). Combination chemotherapy regimens showed numerically longer median OS (RCT combination subgroup median OS (mOS) 12.74 months *vs* monotherapy subgroup 10.60 months; single-arm combination cohort mOS 16.96 months). The most common TRAEs was decreased neutrophil count with an incidence of 24.9%, which was also the most common grade 3 or higher event. The most common irAEs was hypothyroidism (pooled incidence 21.4% in RCTs). Overall toxicity was manageable and safety outcomes were consistent across studies.

**Conclusion:**

Pembrolizumab in conjunction with chemotherapy confers substantial clinical advantage in the management of HER2-negative advanced GC or GEJC, as evidenced by improvements in ORR, OS, and PFS. In contrast, the therapeutic efficacy of pembrolizumab monotherapy appears constrained. Vigilant surveillance for heightened hematologic and gastrointestinal toxicities is warranted within routine clinical practice.

**Systematic Review Registration:**

https://www.crd.york.ac.uk/PROSPERO/, identifier CRD420261298020.

## 1 Introduction

Gastric cancer (GC), including the gastroesophageal junction cancer (GEJC), ranks fourth worldwide in cancer-related mortality and fifth in incidence, with a continuing upward trend that underscores its growing public health burden (1). Early-stage GC is often asymptomatic, and most patients are diagnosed at advanced stages, when treatment efficacy and prognosis are poor (2). The five-year survival rate for advanced GC is only 5~10% (3). *Helicobacter pylori* infection is a strong risk factor and has been classified as a Group 1 carcinogen (4). Other contributors to GC include dietary factors, obesity, smoking, and genetic predisposition. As the disease progresses, the most common clinical manifestations are dyspepsia, anorexia, weight loss, and abdominal pain. In patients with proximal GC or GEJC, symptoms such as dysphagia or reflux may occur (5). Molecular classification of GC partly hinges on the expression of the human epidermal growth factor receptor 2 (HER2) in tumor tissue. HER2, alternatively designated ErbB-2 or ERBB2, constitutes a proto-oncogene mapping to chromosomal locus 17q21. This gene encodes a transmembrane glycoprotein characterized by intrinsic tyrosine kinase enzymatic function. As an integral constituent of the epidermal growth factor receptor family, HER2 mediates downstream signal transduction cascades that govern cellular proliferation and differentiation (6, 7). In Asia, approximately 85% of GCs are HER2-negative, making this the predominant subtype (8, 9). Because these patients’ tumor cells lack HER2 protein expression, they do not benefit from anti-HER2 targeted therapies such as trastuzumab, leaving relatively limited treatment options and unmet clinical needs. Accordingly, focusing on this sizable patient population to develop effective, accessible novel therapeutic strategies has urgent clinical significance.

Immune checkpoint inhibitors (ICIs) target immune-regulatory pathways, disrupting the immune tolerance cycle and allowing T cells to recognize tumor cells. This potentiates the anti-tumor effector function of lymphocytes while counteracting cancer-cell-mediated immunological escape (10, 11). In recent years, immunotherapy has rapidly developed and become a focal point in malignant tumor treatment research, offering new avenues for clinical intervention in patients with non-HER2-positive advanced GC or GEJC (12–14). ICIs have shown promising efficacy in the treatment of these cancers, with key clinical trials such as CheckMate 649 (15) and ORIENT-16 (16) highlighting the efficacy of combining PD-1 inhibitors with chemotherapy. This combination is the standard first-line treatment for HER2-negative advanced GC, including GEJC. Against this backdrop, pembrolizumab, a representative PD-1 inhibitor, has also garnered significant attention for its potential application in this setting. Pembrolizumab is a highly selective humanized IgG4 monoclonal antibody that targets the PD-1 receptor on T cells. By binding to the PD-1 receptor, it blocks its interaction with ligands, thereby alleviating PD-1-mediated immune suppression (17). Due to its early clinical advantages, pembrolizumab has become extensively indicated for the management of diverse solid tumors, such as endometrial cancer (18), triple-negative breast cancer (19), and head and neck squamous cell carcinoma (20).

However, due to the relatively short duration of research on pembrolizumab for the intervention of advanced GC or GEJC and the inconsistent survival benefit outcomes in patients, its efficacy and safety still remain controversial, with a lack of standardized risk assessment systems. Therefore, this study builds upon two previous systematic reviews on pembrolizumab in the treatment of advanced GC and GEJC (21), as well as HER2-positive advanced GC (22), and incorporates insights from earlier studies on the efficacy and safety of PD-1/PD-L1 inhibitors combined with chemotherapy as first-line treatment for unresectable locally HER2-negative advanced GC or GEJC (23). This research further deepens and extends the clinical investigation of pembrolizumab in treating HER2-negative advanced GC or GEJC, aiming to more precisely and specifically validate the efficacy and safety of pembrolizumab in this large group of patients.

## 2 Materials and methods

### 2.1 Registration and protocol

The efficacy and safety of Pembrolizumab in the treatment of HER2-negative advanced gastric or gastroesophageal junction cancer: a systematic review and meta-analysis of emerging clinical data conducted in compliance with the PRISMA 2020 Statement (24, 25) and registered with PROSPERO (Registration Number: CRD420261298020).

### 2.2 Inclusion criteria

1. Timeframe: From the initiation of the library construction until January 31, 2026;
2. Population: Patients with histologically confirmed advanced GC or GEJC, with HER2-negative status;
3. Intervention: The experimental group will receive treatment with pembrolizumab or its combination with other regimens, administered through any route of administration;
4. Control Group: The control group will receive a placebo in combination with or alone with first-line chemotherapy regimens;
5. Outcome Measures: The study must report one or more of the following endpoints: ORR, DoR, OS, PFS, OS-12/24, PFS-12/24, and safety outcomes, including TRAEs, irAEs, etc.;
6. Study Design: Clinical trials must meet the criteria for randomized controlled trials (RCTs) or single-arm studies.

### 2.3 Exclusion criteria

1. Duplicate studies, conference abstracts, and other gray literature;
2. Animal or cell experiments;
3. The control group includes other immunotherapy or targeted therapy agents;
4. Outcome measures do not include ORR, DoR, OS, PFS, OS-12/24, PFS-12/24, TRAEs, irAEs, etc.;
5. Full text cannot be downloaded due to certain limitations, or the article does not provide enough data for a thorough analysis;
6. Trials that are neither RCTs nor single-arm trials.

### 2.4 Search strategy

Our systematic search was conducted across English-language databases, including PubMed, Embase, Web of Science, Scopus, Ovid, Cochrane Library, and CINAHL, as well as the clinical trial registry ClinicalTrials.gov. The search period covered from database inception to 31 January 2026, with a global scope and no language or geographic region restrictions. The search strategy used a mix of controlled vocabulary (subject headings) and free-text terms, adapted to each database’s unique characteristics. Search terms included “advanced gastric cancer or gastroesophageal junction”, “pembrolizumab”, “HER2-negative”, along with “randomized controlled trial” and “single-arm trial”, which were searched in the title and abstract fields. The search syntax was adapted based on the specifications of each database (for the complete search strategy, see **Supplementary Table 1**).

### 2.5 Data extraction and literature screening

The literature identified through specified database search strategies was imported into the NoteExpress software. We conducted the literature screening process in stages according to the PRISMA flowchart: initial content review, followed by abstract and title screening, and finally full-text reading. The collected data include: (1) First author and publication date; (2) Patient characteristics, such as age, sample size, and histological diagnosis; (3) Interventions and treatment protocols for the experimental and control groups; (4) Outcome measures: ORR, DoR, OS, PFS, OS-12/24, PFS-12/24, TRAEs, irAEs, etc.

### 2.6 Risk of bias assessment

The included RCTs had their risk of bias assessed with the Cochrane Risk of Bias tool, version 2.0 (RoB 2) (26). This tool appraises bias across five domains: (1) bias arising from the randomization process; (2) bias due to deviations from intended interventions; (3) bias due to missing outcome data; (4) bias in outcome measurement; and (5) bias in selective reporting of results. Each domain is rated as “low risk”, “some concerns”, or “high risk”. The risk of bias in single-arm studies was assessed using the Risk Of Bias In Non-randomized Studies of Interventions (ROBINS I) tool (27). This instrument evaluates bias across seven domains: (1) bias due to confounding; (2) bias in selection of participants into the study; (3) bias in classification of interventions; (4) bias due to deviations from intended interventions; (5) bias due to missing outcome data; (6) bias in measurement of outcomes; (7) bias in selective reporting of results. Each domain is classified as “low risk”, “moderate risk”, “serious risk”, “critical risk”, or “no information” (28). Two researchers independently assessed the risk of bias in all included RCTs and single-arm studies. The primary outcome measures of this study included ORR, DoR, OS, PFS, OS-12/24, PFS-12/24, TRAEs, and irAEs in patients with HER2-negative advanced GC or GEJC. The above work was independently completed by two researchers (Lingli Jiang and Genming Zhang). In the event of any disagreement, a third researcher (Ke Li) participated in discussions to reach a consensus.

### 2.7 Statistical analysis

All meta-statistical analyses were performed using R software (version 4.5.2, R Foundation for Statistical Computing, Vienna, Austria) with the “meta” package (version 8.2-1) (29) and the “metafor” package (version 4.8-0) (30), amalgamation of figures employing the magick package.

In RCTs with binary outcomes, the effect measure for the between-group comparison of ORR and DoR was the odds ratio (OR) along with its 95% CI, and the Mantel-Haenszel method was applied for pooling. For time-to-event outcomes, including OS, PFS, OS-12/24 and PFS-12/24, the HR and its 95% CI reported in each study were extracted, log-transformed, and then pooled with the inverse variance method. In addition, for the pembrolizumab arm, the pooled proportion and its 95% CI of ORR and adverse event rates were calculated using the logit-transformed inverse variance weighted single-arm proportion pooling approach. Median overall survival (mOS) and median progression-free survival (mPFS) were pooled after natural logarithmic transformation using the inverse variance method, and the results were back-transformed to the original scale expressed in months. In the safety analysis, overall safety indicators such as any grade adverse events, grade 3 or higher adverse events, treatment-related deaths, and discontinuation due to adverse reactions were compared between the pembrolizumab group and the control group using OR. Specific TRAEs and irAEs were grouped according to system organ class (SOC), and the incidence rates in the pembrolizumab arm were summarized with the single-arm proportion pooling method. For multi-arm trials included in the analysis, according to the recommendations of the Cochrane Handbook (31), the treatment arms from the same study were combined before inclusion to avoid double-counting due to a shared control group.

Analytic approach for single-arm studies. ORR and incidence rates of various adverse events were pooled as single-arm proportions with 95%CI using the logit-transformed inverse variance weighting method. MOS and mPFS underwent natural logarithmic transformation; the standard error on the log scale was derived from the 95%CI, pooling was performed with the inverse variance method, and the results were back transformed to the original scale expressed in months. Given the fundamental methodological differences between RCTs and single-arm studies, particularly the absence of concurrent control groups and the inherent risk of confounding in single-arm designs, all of which were judged to be at serious risk of bias according to the ROBINS-I tool, we prespecified that estimates derived from RCTs would serve as the primary evidence for causal inference, whereas estimates from single-arm studies would be interpreted only as exploratory, hypothesis-generating evidence. Any comparisons across study designs are intended for descriptive purposes only and should not be interpreted as implying equivalence of causal effect estimates.

Heterogeneity assessment and model selection. Between-study heterogeneity was evaluated using the Cochran Q test and the I-squared (I^2^) statistic. All analyses reported results from both the common effect model and the random effects model. When substantial heterogeneity was indicated by an I^2^ value greater than 50% or a Q test p-value less than 0.10, the results derived from the random effects model served as the primary reference.

Subgroup analysis and sensitivity analysis. For the RCTs, subgroup analyses of ORR, OS, and PFS were conducted according to PD-L1 combined positive score categories (CPS at least 1 and CPS at least 10), age, region, chemo backbone, and median follow-up. The effect of PD-L1 expression level on treatment efficacy was assessed using the chi- square test across subgroups. For single-arm studies, subgroup analyses of ORR, mOS, and mPFS were similarly performed based on PD-L1 CPS stratification, age, region, chemo backbone, and median follow-up. A stratified sensitivity analysis by treatment regimen (pembrolizumab plus chemotherapy versus pembrolizumab monotherapy) was performed to evaluate the influence of treatment pattern on efficacy and safety outcomes. To assess how individual studies affect the overall results and the robustness of the findings, we performed a leave-one-out sensitivity analysis: each included study was sequentially excluded, and the pooled effect size was recalculated.

Publication bias assessment. For the RCTs, funnel plots were visually inspected for OS, PFS, and ORR. For single-arm studies, funnel plots were visually inspected for ORR, mOS, and mPFS. Because the number of studies included for each outcome was fewer than 10, the Egger linear regression test was not performed following the recommendation of the Cochrane Handbook, and the funnel plot results are provided for reference only.

The risk of bias in RCTs was assessed using the Cochrane RoB 2 tool, which examines five domains: the randomization process, deviations from intended interventions, missing outcome data, outcome measurement, and selective reporting. For single-arm studies, the ROBINS-I tool was applied, covering seven domains: confounding, participant selection, classification of interventions, deviations from intended interventions, missing data, outcome measurement, and selective reporting. Results of the evaluation are displayed as a summary bar chart and a traffic-light plot that illustrates the bias level for each individual item.

## 3 Results

### 3.1 Process of study recognition and selection

**Figure 1:**
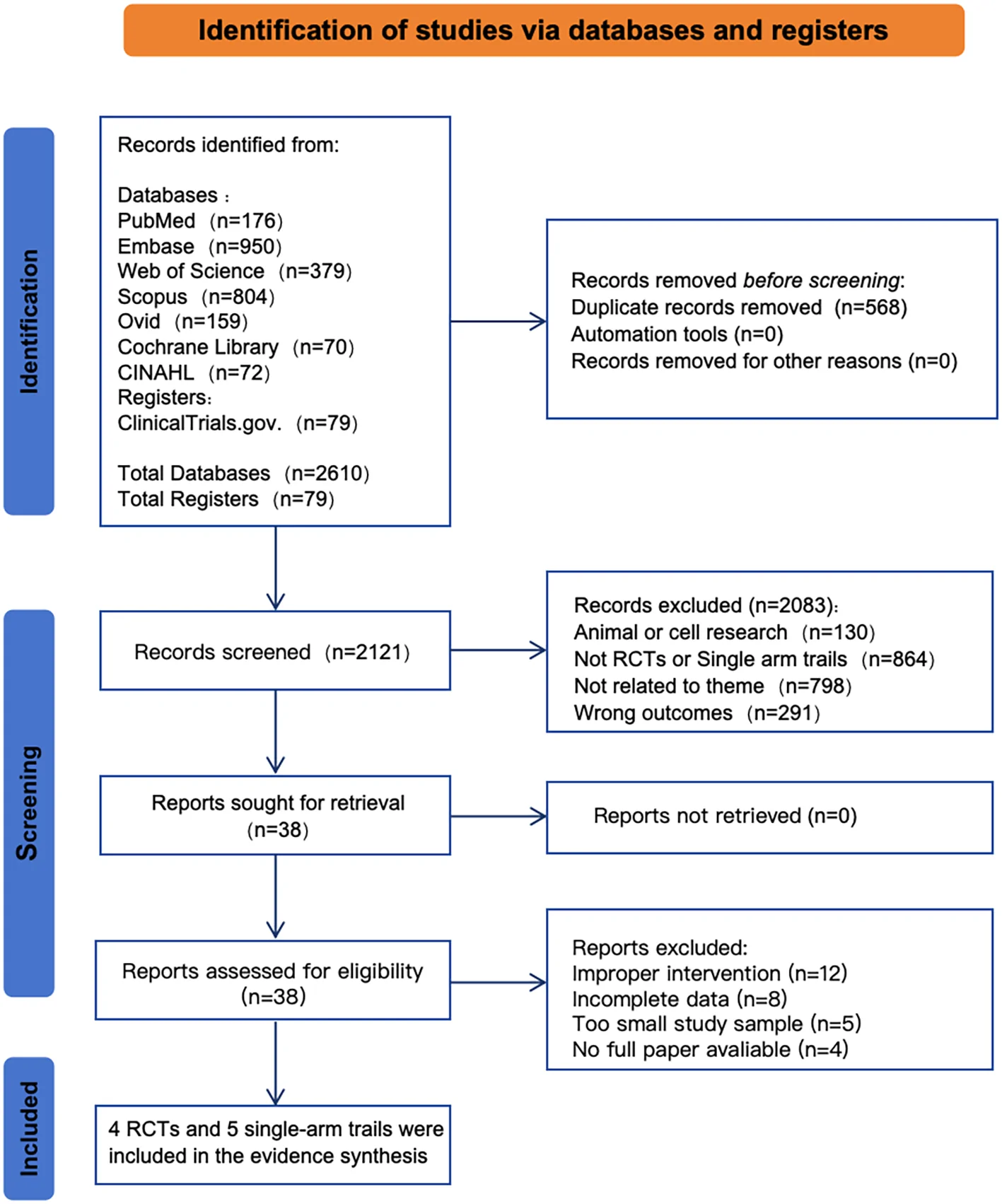
PRISMA diagram of identifying eligible studies.

A systematic search was conducted across multiple databases, including PubMed, Embase, Web of Science, Scopus, Ovid, Cochrane Library, and CINAHL, as well as the clinical trial registry ClinicalTrials.gov, which initially yielded 2689 records. After eliminating 489 duplicate records, a further 2,190 articles were excluded through the first and second screening stages. Ultimately, nine trials (32–38) from seven articles, comprising four RCTs (32–34) and five single-arm studies (35–38), were included in the meta-analysis. The screening procedure is presented in **Figure 1**.

### 3.2 General study characteristics

The sample sizes of the nine included studies ranged from 25 to 1579. A total of 3406 patients with HER2-negative advanced GC or GEJC were enrolled, with ages spanning from 20 to 87 years and median follow-up durations ranging from 13.8 to 32.2 months. All studies adopted the World Health Organization Response Evaluation Criteria in Solid Tumors (RECIST version 1.1) to assess treatment response. Multiple countries served as the settings for the investigation, including Japan, Korea, the United Kingdom, and China. The outcome measures reported across studies included ORR, DoR, OS, PFS, OS-12/24, PFS-12/24, and safety outcomes comprising TRAEs and irAEs. The baseline characteristics and quality assessment results of the included studies are presented in **Table 1**.

**Table 1:** Baseline characteristics of included studies.

Country	First author	Year	Registration number	Study design	Phase	Research scale	Pembro(N)	Control(N)	Pembrolizumab therapy	Pembro dose and schedule	Control therapy	Molecular subtype	Median age of Pembro group (years)	Median age of control group (years)	Median follow-up time (months)	Outcomes
Korea	Sun Young Rha (32)	2023	KEYNOTE-859	RCT	III	Multicenter	790	789	pembrolizumab plus chemotherapy	Pembro (200mg IV 21-day cycle)	placebo plus chemotherapy	HER2 -	61(52-67)	62(52-69)	31	ORR, DoR, OS, PFS, AEs, irAEs, OS-12, OS-24, PFS-12, PFS-24
Japan	Kohei Shitara (33)	2020	KEYNOTE-062	RCT	III	Multicenter	256	250	single-agent pembrolizumab	Pembro (200mg IV 21-day cycle)	placebo plus chemotherapy	HER2 -	61(20-83)	62.5(23-87)	29.4	ORR, DoR, OS, PFS, AEs, irAEs, OS-12, OS-24
Japan	Kohei Shitara (33)	2020	KEYNOTE-062	RCT	III	Multicenter	257	250	pembrolizumab plus chemotherapy	Pembro (200mg IV 21-day cycle)	placebo plus chemotherapy	HER2 -	62(22-83)	62.5(23-87)	29.4	ORR, DoR, OS, PFS, AEs, irAEs, OS-12, OS-24
Japan	Kohei Shitara (34)	2025	LEAP-015	RCT	III	Multicenter	443	437	pembrolizumab plus lenvatinib plus chemotherapy	Pembro (400 mg IV, 42-day cycle)	single-agent chemotherapy	HER2 -	62(21-84)	61(24-84)	32.2	ORR, DoR, OS, PFS, AEs, irAEs, OS-12, OS-24, PFS-12, PFS-24
Korea	Yung‐Jue Bang (35)	2019	KEYNOTE-059	Single-arm	II	Multicenter	25	NA	single-agent pembrolizumab	Pembro (200mg IV 21-day cycle)	NA	HER2 -	64(21-82)	NA	13.8	ORR, DoR, OS, PFS, AEs, irAEs, OS-12, OS-24, PFS-12
Korea	Yung‐Jue Bang (35)	2019	KEYNOTE-059	Single-arm	II	Multicenter	31	NA	single-agent pembrolizumab	Pembro (200mg IV 21-day cycle)	NA	HER2 -	62(32-75)	NA	17.5	ORR, DoR, OS, PFS, AEs, irAEs, OS-12, PFS-12
Japan	Akihito Kawazoe (36)	2020	KEYNOTE-659	Single-arm	II	Multicenter	54	NA	single-agent pembrolizumab	Pembro (200mg IV 21-day cycle)	NA	HER2 -	66(32-75)	NA	16.9	ORR, DoR, OS, PFS, AEs, OS-12, OS-24, PFS-12, PFS-24
Japan	Kensei Yamaguchi (37)	2022	KEYNOTE-659	Single-arm	II	Multicenter	46	NA	single-agent pembrolizumab	Pembro (200mg IV 21-day cycle)	NA	HER2 -	65(30-75)	NA	17.1	ORR, DoR, OS, PFS, AEs, OS-12, OS-24, PFS-12, PFS-24
London	Ian Chau (38)	2020	NCT02443324	Single-arm	I a/b	Multicenter	28	NA	pembrolizumab plus ramucirumab	Pembro (200mg IV 21-day cycle)	NA	HER2 -	63(31-83)	NA	26.4	ORR, DoR, OS, PFS, AEs, irAEs, OS-12, PFS-12

### 3.3 Quality assessment

**Figure 2:**
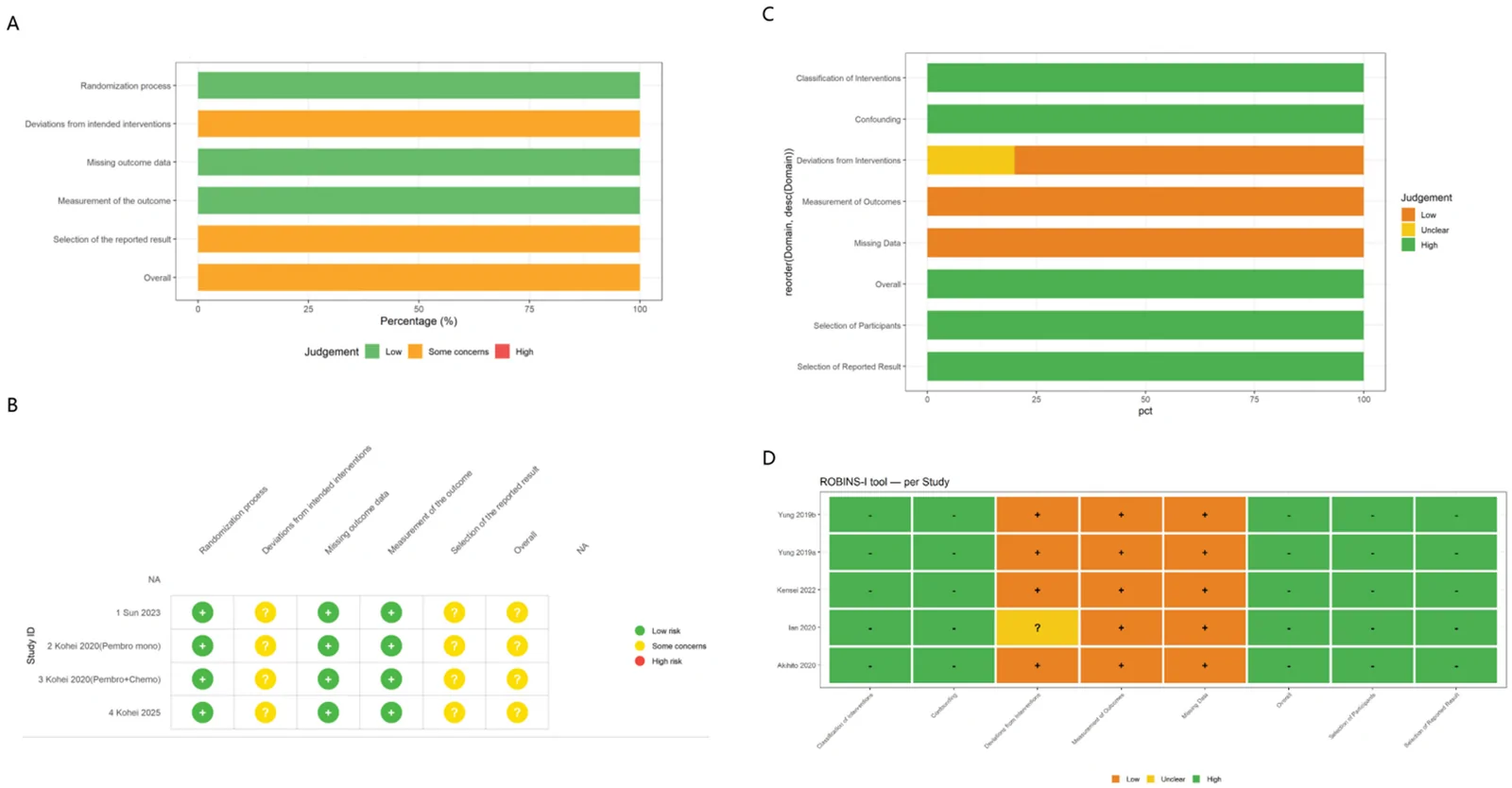
Quality assessment of enrolled studies. **(A)** Overall quality assessment of 4 RCTs using Revised cochrane risk of bias tool. **(B)** Detailed assessment of the risk of bias for RCTs. **(C)** Overall quality assessment of single-arm studies via the ROBINS-I tool. **(D)** Detailed assessment of the risk of bias for single arm studies.

On the whole, the four RCTs and five single-arm studies provided fairly thorough quality assessment reports. Regarding the four RCTs, the overall risk of bias received a moderate rating (**Figure 2A**). Of these, one RCT adopted an open-label design, which led to a judgment of some concerns regarding its randomization process. **Figure 2B** provides a detailed breakdown of the bias risk assessment for every study. For the included single-arm studies, the ROBINS I tool was used to assess the risk of bias. The five single-arm studies in this analysis lacked a concurrent control group. According to the judgment logic of the ROBINS I tool, such a design carries an inherent and uncorrectable serious flaw in the domain of confounding bias. Consequently, all single-arm studies were rated as having a high risk of bias. This likely reflects the methodological limitation of the current body of evidence rather than the poor execution quality of individual studies. The summarized ROBINS I assessment results for each study are shown in **Figures 2C, D**.

### 3.4 Meta-analysis results

All 9 studies provided data on the primary efficacy endpoints: ORR, DoR, OS, PFS, OS-12/24, PFS-12/24, and adverse events. Recognizing that RCTs and single-arm studies are methodologically distinct, we performed meta-analysis stratified by study type. It should be noted that for the analysis of multi-arm RCTs presented in the following results, we followed the Cochrane Handbook recommendations for handling multi-arm trials by combining multiple treatment arms from the same study into a single group for analysis. For single-arm studies containing multiple cohorts, we analyzed each cohort separately.

#### 3.4.1 Objective response rate

**Figure 3:**
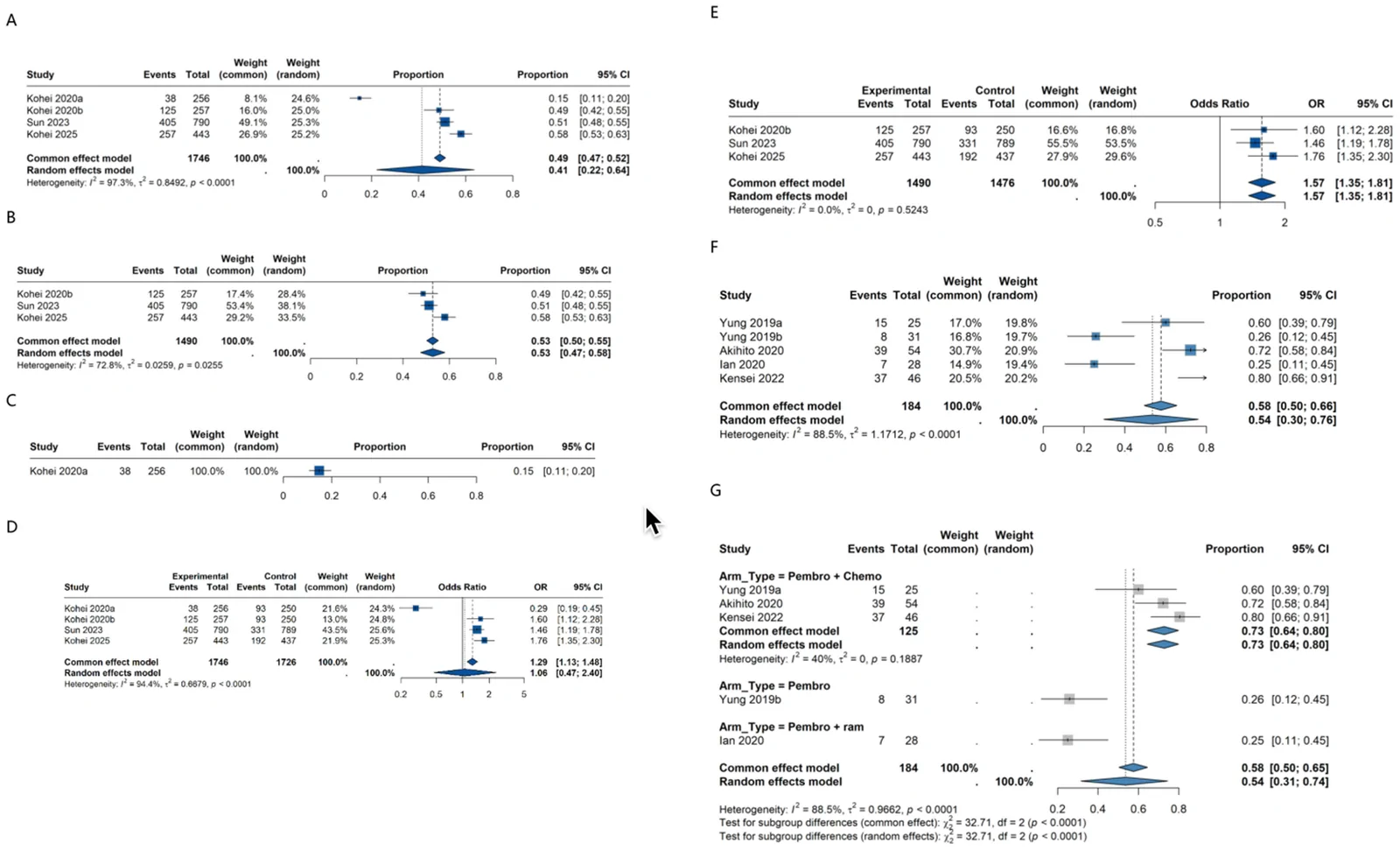
Pooled ORR of Pembrolizumab in GC or GEJC patients **(A)** Pooled ORR main of RCTs; **(B)** Pooled ORR sens chemo of RCTs; **(C)** Pooled ORR sens mono of RCTs; **(D)** ORR OR main of RCTs; **(E)** ORR OR sens chemo of RCTs; **(F)** Pooled ORR of single-arms; **(G)** ORR by ArmType of single-arms.

All four RCTs included in the analysis reported ORR. Among them, Kohei 2020 was a multi-arm trial containing two treatment arms of pembrolizumab plus chemotherapy and pembrolizumab monotherapy. A total of 1746 patients received pembrolizumab-based regimens. The pooled ORR based on a random effects model was 41% (95%CI:22%-64%), with substantial between-study heterogeneity (I²=97.3%, τ²=0.8492, p<0.0001) (**Figure 3A**). Sensitivity analysis showed (**Figure 3B**) that in the subgroup receiving pembrolizumab plus chemotherapy (3 studies, n=1490), the pooled ORR was 53% (95%CI, 47%-58%; I²=72.8%, p=0.0255). For pembrolizumab monotherapy, only one study (n=256) was available for analysis, yielding an ORR of 15% (95%CI: 11%-20%). These findings indicate that the response rate of the combination chemotherapy regimen was significantly higher than that of the monotherapy regimen (**Figure 3C**).

For the between-group comparison using the control group as the reference, the pooled odds ratio for ORR in the pembrolizumab group was 1.06 (95%CI:0.47-2.40). The difference did not reach statistical significance, and substantial between-study heterogeneity was observed (I²=94.4%, τ²=0.6679, p<0.0001) (**Figure 3D**). Of note, when the sensitivity analysis was restricted to studies of pembrolizumab plus chemotherapy (**Figure 3E**), the pooled odds ratio increased to 1.57 (95%CI:1.35-1.81), achieving statistical significance, and between-study heterogeneity was completely eliminated (I²=0%, p=0.52), indicating high consistency across the three studies. This finding suggests that the primary source of heterogeneity in the main analysis was the inclusion of the pembrolizumab monotherapy arm from Kohei 2020, which had an odds ratio of 0.79 (95%CI:0.57-1.08), a direction favoring the control group and opposite to that of the combination chemotherapy arms. Consequently, this arm lowered the overall effect estimate and substantially increased heterogeneity. Integrating the results from **Figure 3**, the pembrolizumab plus chemotherapy regimen not only achieved a high absolute response rate (pooled ORR of 53%) but also significantly improved the likelihood of objective response compared with chemotherapy alone (odds ratio of 1.57). In contrast, pembrolizumab monotherapy did not demonstrate a trend toward superiority over the control group in terms of ORR.

Five single-arm studies derived from four independent trials, with Yung 2019 including two cohorts of pembrolizumab plus chemotherapy and pembrolizumab monotherapy, enrolled a total of 184 patients and also reported ORR. Based on a random effects model, the pooled ORR for pembrolizumab was 54% (95%CI:30%-76%), with substantial between-study heterogeneity (I²=88.5%, τ²=1.1712, p<0.0001) (**Figure 3F**). Subgroup analysis stratified by treatment regimen (**Figure 3G**) showed that for the pembrolizumab plus chemotherapy cohort (3 studies, n=125), the pooled ORR was 73% (95%CI:64%-80%), with markedly reduced heterogeneity (I²=40%, p=0.19), indicating good consistency across studies in this subgroup. For the pembrolizumab monotherapy cohort (Yung 2019b, n=31), the ORR was 26% (95%CI:12%-45%). For the pembrolizumab plus ramucirumab cohort (Ian 2020, n=28), the ORR was 25% (95%CI:11%-45%). The two regimens showed similar efficacy, and both were significantly lower than that of the combination chemotherapy regimen. The difference between subgroups was statistically significant (χ²=32.71, degrees of freedom=2, p<0.0001), indicating that treatment modality was the primary source of heterogeneity. The findings from the single-arm studies were consistent with the trends observed in the RCT sensitivity analysis. The response rate of the combination chemotherapy regimen (53% in RCTs and 73% in single-arm studies) was substantially higher than that of the monotherapy regimen (15% in RCTs and 26% in single-arm studies). This further supports the clinical value of pembrolizumab plus chemotherapy as the preferred treatment strategy for HER2-negative advanced GC or GEJC.

#### 3.4.2 Overall survival

**Figure 4:**
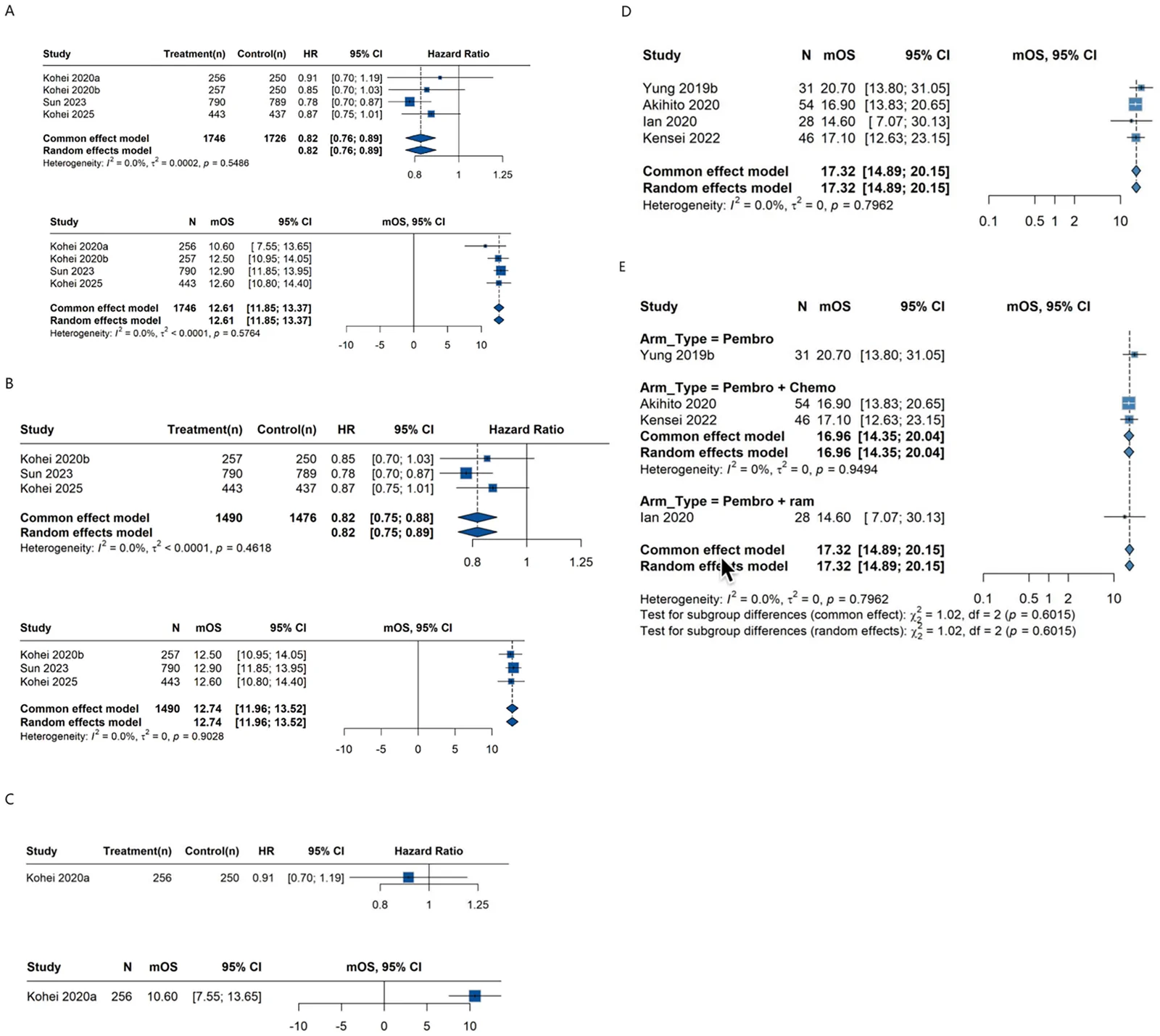
Pooled mOS of included studies. **(A)** Combined OS main of RCTs; **(B)** Combined OS sens chemo of RCTs; **(C)** Combined OS sens mono of RCTs; **(D)** Pooled mOS of single-arms; **(E)** mOS by ArmType of single-arms.

All four RCTs (including the two treatment arms from the multi-arm trial Kohei 2020) reported OS outcomes. In the HR analysis, the pembrolizumab group showed a significant survival benefit over the control group, with a pooled HR of 0.82 (95%CI: 0.76-0.89) and no between-study heterogeneity (I²=0%, p=0.55). The pooled mOS in the pembrolizumab-treated group was 12.61 months (95%CI:11.85-13.37 months), with highly consistent results across studies (I²=0%, p=0.73) (**Figure 4A**).

Sensitivity analysis restricted to the pembrolizumab plus chemotherapy arms (3 studies, treatment group n=1490) yielded a pooled HR of 0.82 (95%CI:0.75-0.89) and a pooled mOS of 12.74 months (95%CI:11.96-13.52 months). Both estimates showed no heterogeneity (I²=0%), consistent with the main analysis and indicating good robustness of the overall results (**Figure 4B**). For the pembrolizumab monotherapy arm, only the Kohei 2020 study was available for analysis (treatment group n=256), with an HR of 0.91 (95%CI: 0.70-1.19); the confidence interval crossed 1, indicating no statistical significance. The mOS was 10.60 months (95%CI:7.55-13.65 months), approximately two months shorter than that of the combination chemotherapy regimen (**Figure 4C**).

Among the single-arm studies, four cohorts derived from three independent trials (159 patients in total) reported median OS with complete confidence intervals. The combination chemotherapy cohort of Yung 2019 (n=25) reported mOS, but the upper bound of its confidence interval was not estimable (NE), suggesting that the survival benefit of this cohort might not have been fully realized. This cohort was not included in the quantitative pooling because the standard error on the log scale could not be calculated. The pooled mOS was 17.32 months (95%CI:14.89-20.15 months), with no between-study heterogeneity (I²=0%, p=0.80) (**Figure 4D**). Subgroup analysis by treatment regimen (**Figure 4E**) showed that for the pembrolizumab plus chemotherapy cohort (2 studies, n=100), the pooled mOS was 16.96 months (95%CI:14.35-20.04 months; I²=0%). For the pembrolizumab monotherapy cohort (Yung 2019b, n=31), the mOS was 20.70 months (95%CI: 13.80-31.05). For the pembrolizumab plus ramucirumab cohort (Ian 2020, n=28), the mOS was 14.60 months (95%CI: 7.07-30.13). The difference among the three subgroups was not statistically significant (subgroup difference test χ²=1.02, degrees of freedom=2, p=0.60). Of note, the pembrolizumab monotherapy cohort had the numerically longest mOS, which may be attributable to the enrollment of an immunologically favorable population selected by specific biomarkers in that cohort, rather than indicating that monotherapy is superior to combination therapy. Methodologically, this phenomenon primarily stems from the inherent lack of a concurrent control group in single-arm studies, which results in a very high risk of confounding bias according to the ROBINS-I assessment. Statistically, such bias may potentially influenced by an overestimation of pooled survival outcomes in single-arm cohorts, with a pooled mOS of 17.32 months, which appears superior to that reported in RCT cohorts with more stringent eligibility criteria (12.64 months). In the absence of concurrent controls and quantitative bias adjustment, the observed improvement in efficacy outcomes from single-arm cohorts cannot be clearly distinguished from the confounding effects of selection bias, such as better baseline performance status and limited sample representativeness. Therefore, this apparent numerical advantage may be at least partially, or even predominantly, attributable to systematic differences in patient selection rather than solely reflecting the intrinsic efficacy of the treatment strategy. Accordingly, cautious interpretation is warranted when extrapolating these findings to real-world clinical practice.

#### 3.4.3 Progression-free survival

**Figure 5:**
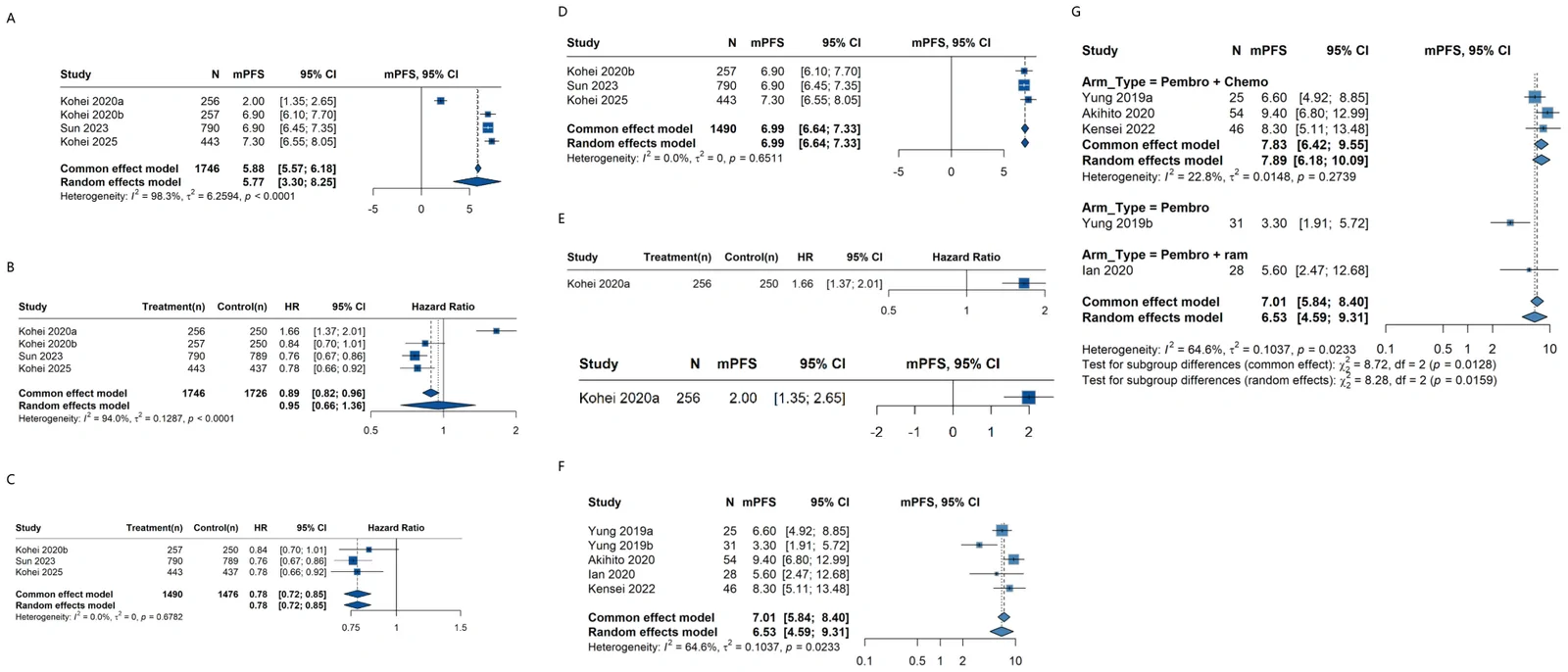
Pooled mPFS of Pembrolizumab in GC or GEJC patients. **(A)** Combined PFS main A of RCTs; **(B)** Combined PFS main B of RCTs; **(C)** Combined PFS sens chemo A of RCTs; **(D)** Combined PFS sens chemo B of RCTs; **(E)** Combined PFS sens mono of RCTs; **(F)** Pooled mPFS of single-arms; **(G)** mPFS by ArmType of single-arms.

All four RCTs reported PFS outcomes for patients with HER2-negative advanced GC or GEJC treated with pembrolizumab (presented as three analytical entries after handling the multi-arm trial according to the Cochrane Handbook). The pooled mPFS in the pembrolizumab group was 6.77 months (95%CI: 6.19-7.36 months), with moderate between-study heterogeneity (I²=69.1%, τ²=0.1851, p=0.04) (**Figure 5A**). In the HR analysis, the pooled HR from the main analysis was 0.89 (95%CI: 0.67-1.17). Under the random effects model, the CI crossed 1, indicating no statistical significance, and between-study heterogeneity was extremely high (I²=92.2%, τ²=0.0549, p<0.0001), suggesting marked differences in the direction of PFS effects across studies (**Figure 5B**).

Sensitivity analysis revealed the primary source of heterogeneity. When the analysis was restricted to studies of pembrolizumab plus chemotherapy (3 studies, treatment group n=1490), the pooled HR decreased to 0.78 (95%CI: 0.72-0.85), with high statistical significance and complete elimination of between-study heterogeneity (I²=0%, p=0.68) (**Figure 5C**). The corresponding pooled mPFS was 6.99 months (95%CI: 6.64-7.33 months, I²=0%), indicating highly consistent results across studies and that the combination chemotherapy regimen reduced the risk of PFS disease progression by approximately 22% (**Figure 5D**). In contrast, for the pembrolizumab monotherapy arm (Kohei 2020, n=256), the HR was 1.66 (95%CI: 1.37-2.01), a direction favoring the chemotherapy control group, with a mPFS of only 2.00 months (95%CI: 1.35-2.65), markedly shorter than the 6.99 months observed with the combination chemotherapy regimen (**Figure 5E**). This finding suggests that in the unselected population of HER2-negative advanced GC or GEJC, pembrolizumab monotherapy has limited disease control efficacy. This also explains the extremely high heterogeneity observed in the main analysis, namely that the negative effect of the monotherapy arm in the Kohei 2020 study counterbalanced the positive effect of the combination chemotherapy arms, leading to a distorted overall estimate.

Five single-arm study cohorts (n=184) reported PFS outcomes. The pooled mPFS was 6.53 months (95%CI: 4.59-9.31), with moderate between-study heterogeneity (I²=64.6%, τ²=0.1037, p=0.023) (**Figure 5F**). Subgroup analysis stratified by treatment regimen (**Figure 5G**) explained the source of this heterogeneity (subgroup difference test χ²=8.28, p=0.016). The pembrolizumab plus chemotherapy cohort (3 studies, n=125) had the longest pooled mPFS of 7.89 months (95%CI: 6.18-10.09), with low within-subgroup heterogeneity (I²=22.8%, p=0.27). The pembrolizumab plus ramucirumab cohort (Ian 2020, n=28) had an mPFS of 5.60 months (95%CI: 2.47-12.68). The pembrolizumab monotherapy cohort (Yung 2019b, n=31) had the shortest mPFS of only 3.30 months (95%CI: 1.91-5.72). This result is consistent with the trend observed in the RCT sensitivity analysis, namely that the combination chemotherapy regimen was significantly superior to monotherapy in prolonging PFS, further confirming that treatment modality is a key factor influencing the antitumor activity of pembrolizumab. Similarly, given the extremely high risk of confounding bias identified in single-arm studies by the ROBINS-I assessment, this methodological limitation may also have clinically relevant implications for PFS outcomes. However, without appropriate adjustment for potential confounders, the possibility that these findings were influenced by selection bias, such as differences in baseline prognostic characteristics, cannot be excluded. Therefore, a causal relationship between the observed outcomes and the direct therapeutic effects of the treatment strategy cannot be established in the present study. Accordingly, this finding should be interpreted as exploratory evidence rather than definitive evidence when considering its translation into clinical practice.

#### 3.4.4 Long-term survival and durable remission as clinical endpoints

**Figure 6:**
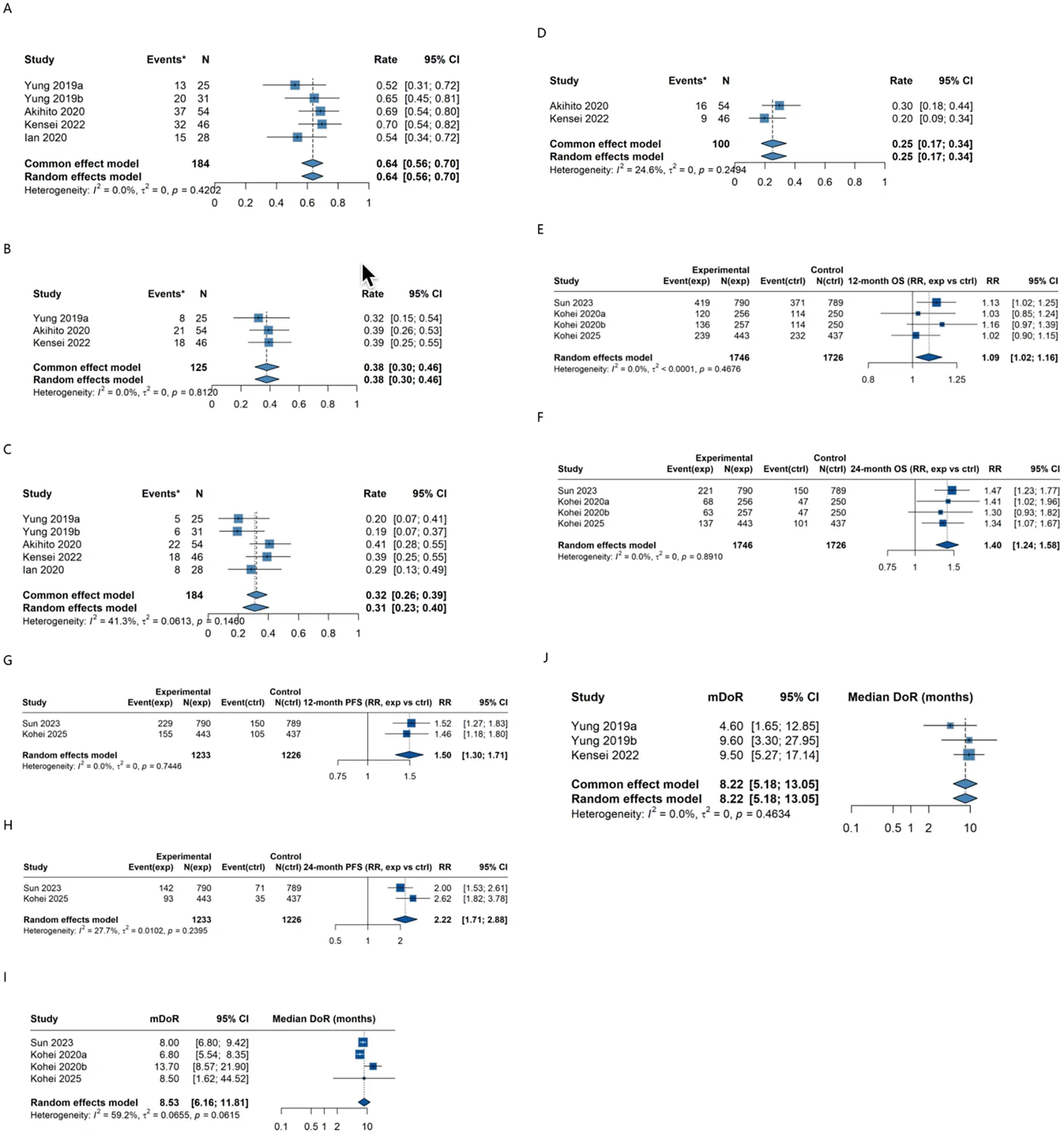
Landmark survival rates and duration of response of included studies. **(A)** 12-month OS rate of single-arms; **(B)** 24-month OS rate of single-arms; **(C)** 12-month PFS rate of single-arms; **(D)** 24-month PFS rate of single-arms; **(E)** 12-month OS RR of RCTs; **(F)** 24-month OS RR of RCTs; **(G)** 12-month PFS RR of RCTs; **(H)** 24-month PFS RR of RCTs; **(I)** DoR of RCTs; **(J)** DoR of single-arms.

To more comprehensively evaluate the long-term efficacy of immune-based combination therapy, we supplemented our primary analysis by extracting landmark survival rates and DoR from the included studies. The relevant results are presented in the supplementary figures. Specifically, landmark survival rates in single-arm cohorts are shown in (**Figures 6A–D**), relative landmark benefits in RCTs are shown in (**Figures 6E–H**), DoR analyses are shown in (**Figures 6I, J**). In the pooled single-arm cohorts, the 12-month and 24-month OS rates were 0.64 (95%CI: 0.56-0.70) and 0.38 (95%CI: 0.3-0.46), respectively, and the corresponding PFS rates were 0.31 (95%CI: 0.23-0.4) and 0.25 (95%CI: 0.17-0.34), with low to moderate heterogeneity (I²≤41.3%). In RCTs, the RR for landmark OS favored the experimental arm over the control arm, increasing from 1.09 (95%CI: 1.02-1.16) at 12 months to 1.40 (95%CI: 1.24-1.58) at 24 months. Similarly, the RR for PFS increased from 1.50 (95%CI: 1.30-1.71) to 2.22 (95%CI: 1.71-2.88) over the same period, with low heterogeneity across all analyses (I²≤27.7%). This temporal widening of relative benefit is consistent with the characteristic “long-tail effect” of immunotherapy, wherein survival advantages become more pronounced with extended follow-up. In addition, the pooled median DoR was similar in RCTs (8.53, 95%CI: 6.16-11.81) and single-arm cohorts (8.22, 95%CI: 5.18-13.05), suggesting that the durability of response associated with this therapeutic strategy is reproducible across different study designs.

#### 3.4.5 Treatment-related adverse events

**Figure 7:**
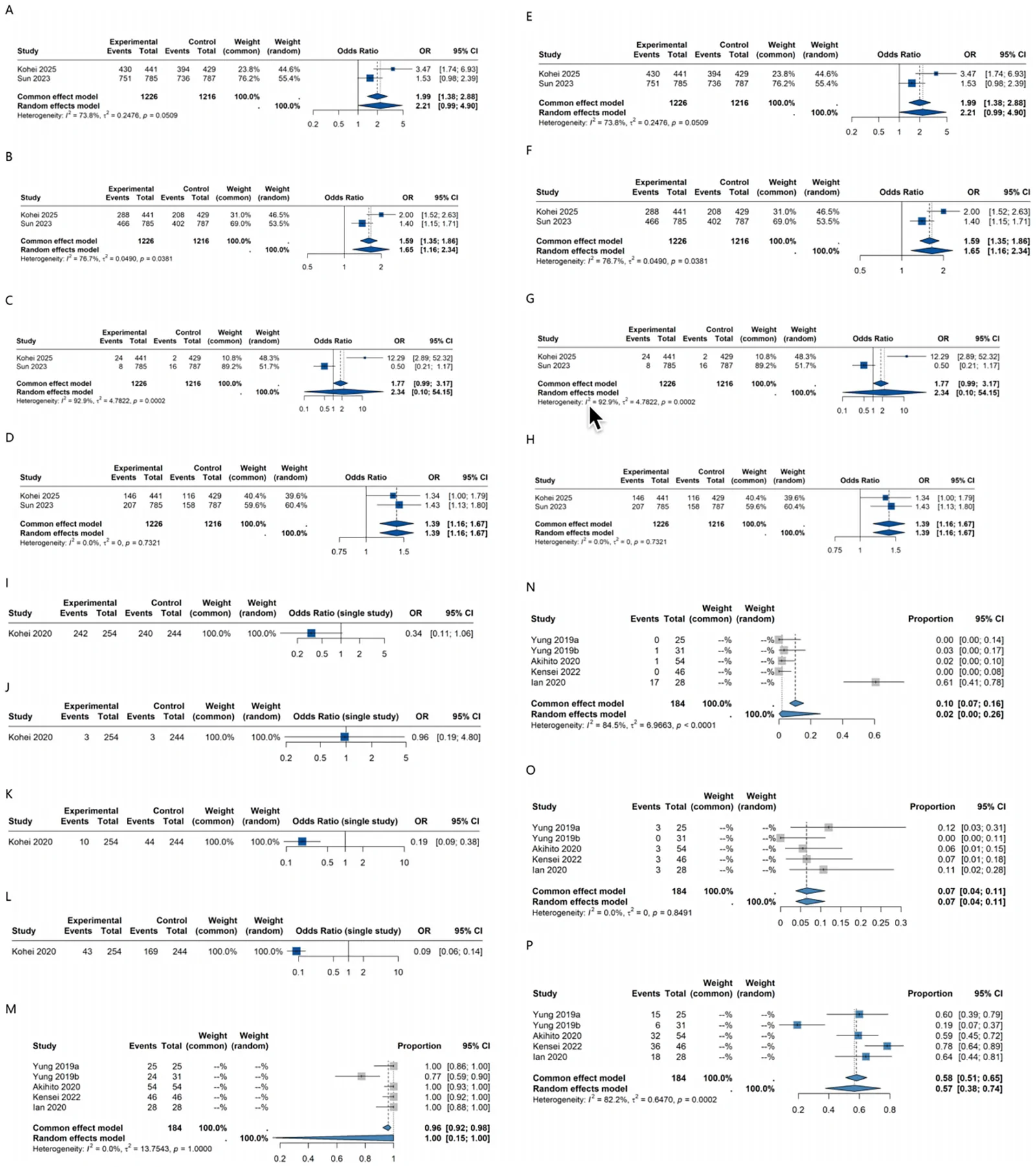
TRAEs and irAEs of included studies. **(A)** AnyAE main of RCTs; **(B)** Grade35 main of RCTs; **(C)** Death main of RCTs; **(D)** Discont main of RCTs; **(E)** AnyAE sens chemo of RCTs; **(F)** Grade35 sens chemo of RCTs; **(G)** Death sens chemo of RCTs; **(H)** Discont sens chemo of RCTs; **(I)** AnyAE sens mono of RCTs; **(J)** Grade35 sens mono of RCTs; **(K)** Death sens mono of RCTs; **(L)** Discont sens mono of RCTs; **(M)** AnyAE of single-arms; **(N)** Death of single-arms; **(O)** Discont of single-arms; **(P)** Grade35 AE of single-arms;.

TRAEs were summarized in both RCTs and single-arm studies. The RCTs component included three studies, with 1730 patients in the pembrolizumab group and 1460 patients in the control group. In the overall primary analysis, the incidence rates of any grade TRAEs were 96.4% (1667/1730) in the pembrolizumab group and 93.8% (1370/1460) in the control group, with a pooled odds ratio of 2.21 (95%CI: 0.99-4.90, I^2^ = 73.8%, p=0.05) (**Figure 7A**). The incidence rates of grade 3 or higher TRAEs were 56.6% (980/1730) and 53.4% (779/1460), respectively, with a pooled odds ratio of 1.65 (95%CI: 1.16-2.34, I^2^ = 76.7%, p=0.04) (**Figure 7B**). Treatment-related deaths accounted for 2.3% (40/1730) and 1.4% (21/1460), respectively, with a pooled odds ratio of 2.34 (95%CI: 0.10-54.15, I^2^ = 92.9%, p=0.0002) (**Figure 7C**). Discontinuation due to adverse reactions occurred in 25.0% (432/1730) and 21.8% (318/1460), respectively, with a pooled odds ratio of 1.39 (95%CI: 1.16-1.67, I^2^ = 0.00%, p=0.73) (**Figure 7D**).

Sensitivity analysis stratified by treatment regimen showed that in the pembrolizumab plus chemotherapy subgroup (n=1476 *vs* 1460), the pooled odds ratios for any grade TRAEs, grade 3 or higher TRAEs, treatment related death, and discontinuation due to adverse reactions were 2.21 (95%CI: 0.99-4.90, I^2^ = 73.8%), 1.65 (95%CI: 1.16-2.34, I^2^ = 76.7%), 2.34 (95%CI: 0.10-54.15, I^2^ = 85.9%), and 1.39 (95%CI: 1.16-1.67, I^2^ = 0.0%) respectively(**Figures 7E–H**). In the pembrolizumab monotherapy subgroup (only Kohei 2020, n=254 *vs* 244), the odds ratios for the above four indicators were 0.34 (95%CI: 0.11-1.06), 0.96 (95%CI:0.19-4.80), 0.19 (95%CI: 0.09-0.38), and 0.09 (95%CI: 0.06-0.14), respectively (**Figures 7I–L**).

Pooled incidence rates of specific treatment-related adverse events categorized by system organ class are presented in **Table 2** (complete data available in **Supplementary Table 2**). Among hematologic toxicities, neutropenia (24.9%, 95%CI:10.3%-49.0%) and anemia (24.7%, 95%CI: 19.0%-31.4%) were the most common. Gastrointestinal toxicities mainly included nausea (36.9%, 95%CI: 30.6%-43.6%), diarrhea (27.6%, 95%CI: 15.8%-43.7%), and decreased appetite (22.9%, 95%CI: 17.4%-29.6%). Other frequently observed toxicities were fatigue (21.8%, 95%CI: 19.6%-24.1%), hand foot syndrome (18.6%, 95%CI: 11.9%-27.8%), elevated aspartate aminotransferase (17.9%, 95%CI: 15.9%-20.2%), and hypothyroidism (15.3%, 95%CI: 7.7%-28.2%). Most adverse events showed high between-study heterogeneity with I^2^ exceeding 75%; detailed heterogeneity statistics are provided in **Supplementary Table 3**.

**Table 2:** Pooled incidence of common treatment-related adverse events (pooled rate ≥5%) in patients receiving pembrolizumab-based regimens.

System/adverse event	No. of studies (k)	Events/total	Pooled rate (%)	95% CI	I² (%)	p (Q test)
Hematologic						
Neutrophil count decreased	3	454/1730	24.9	10.3–49.0	98.5	<0.001
Anemia	3	447/1730	24.7	19.0–31.4	90.1	<0.001
Neutropenia	2	230/1289	17.8	15.8–20.0	0	0.774
Decreased platelet count	3	338/1730	15.7	5.2–38.9	97.4	<0.001
WBC decreased	3	235/1730	12.7	5.9–25.2	96.2	<0.001
Thrombocytopenia	2	110/1289	7.7	3.9–14.6	90.3	0.001
Gastrointestinal						
Nausea	3	650/1730	36.9	30.6–43.6	87.2	<0.001
Diarrhea	3	500/1730	27.6	15.8–43.7	96.8	<0.001
Decreased appetite	3	392/1730	22.9	17.4–29.6	88.1	<0.001
Vomiting	3	369/1730	19.7	13.5–27.9	93.8	<0.001
Stomatitis	2	99/945	10.2	4.8–20.2	92.7	<0.001
Mucosal inflammation	2	94/945	10	7.4–13.4	58.3	0.121
Constipation	2	68/945	7.1	4.1–12.1	81.3	0.021
Dysgeusia	2	49/945	5.2	4.0–6.8	0	0.531
Skin						
PPE syndrome	3	343/1730	18.6	11.9–27.8	93.2	<0.001
Rash	2	87/945	9.2	7.5–11.3	0	0.444
Pruritus	2	80/945	8.5	6.8–10.7	16	0.275
Constitutional						
Fatigue	3	375/1730	21.8	19.6–24.1	17.2	0.299
Asthenia	3	178/1730	9.9	6.8–14.1	81	0.005
Weight decreased	2	95/945	9.1	2.8–26.1	96.4	<0.001
Hepatic						
AST increased	2	220/1226	17.9	15.9–20.2	0	0.772
ALT increased	2	171/1226	14.2	11.5–17.3	52.8	0.145
Bilirubin increased	2	113/1226	9.2	7.4–11.3	25.6	0.246
Endocrine						
Hypothyroidism	3	273/1730	15.3	7.7–28.2	96.7	<0.001
Neurologic						
Peripheral neuropathy	3	245/1730	11.9	5.3–24.6	95.5	<0.001
Peripheral sensory neuropathy	3	224/1730	11.4	6.5–19.5	93.5	<0.001

A total of 5 single-arm studies were included, comprising 184 patients. The pooled incidence rate of any grade TRAEs was 0.96 (common effect model, 95%CI: 0.92-0.98, I^2^ = 0.0%). The incidence rate was 1.00 in 4 studies and only 0.77 in Yung 2019b. The pooled incidence rate of treatment-related death was 0.02 (95%CI: 0.00-0.26, I^2^ = 84.5%, p<0.0001). Notably, Ian 2020 reported 17 out of 28 cases (0.61), representing a major outlier, while the remaining 4 studies each reported no more than 1 case. The pooled incidence rate of discontinuation due to adverse reactions was 0.07 (95%CI: 0.04-0.11, I^2^ = 0.0%, p=0.849), showing good consistency across studies. The pooled incidence rate of grade 3 or higher TRAEs was 0.57 (95%CI: 0.38-0.74, I^2^ = 82.2%, p=0.0002), with substantial variation across studies ranging from 0.19 to 0.78, respectively(**Figures 7M–P**).

After stratification by treatment regimen, in the combination chemotherapy subgroup (4 studies, 153 patients), the incidence of any grade TRAEs was 100%, that of grade 3 or higher TRAEs was 0.66 (95%CI: 0.57-0.74, I^2^ = 33.0%), treatment related death was 0.01 (95%CI: 0.0-0.46, I^2^ = 81.9%), and discontinuation due to adverse events was 0.08 (95%CI: 0.05-0.13, I^2^ = 0.0%). In the monotherapy subgroup, only one study (Yung 2019b, 31 patients) was available, with grade 3 or higher TRAEs of 19.4%, treatment-related death of 3.2%, and discontinuation due to adverse events of 0%. Compared with the primary analysis, the heterogeneity for grade 3 or higher TRAEs in the combination chemotherapy subgroup decreased from an I^2^ of 82.2% to 33.0%, suggesting that the high heterogeneity observed in the primary analysis was partly attributable to the differing toxicity profiles between monotherapy and combination regimens (**Supplementary Figures 1**–**4**).

TRAEs with a pooled incidence of 5% or higher are presented by system organ class in **Supplementary Table 4**. Complete details of all TRAEs are provided in **Supplementary Table 2**. Among hematologic toxicities, neutropenia had the highest pooled incidence at 72.3% (95%CI:47.6%-88.2%, k=3, I^2^ = 89.4%, p<0.001), followed by thrombocytopenia 36.4% (95%CI:22.5%-53.0%, I^2^ = 78.9%, p=0.009), anemia 23.0% (95%CI: 15.4%-32.8%, I^2^ = 44.8%, p=0.163), and leukopenia 16.0% (95%CI: 10.6%-23.5%, I^2^ = 0.0%, p=0.671). For gastrointestinal toxicities, the predominant events were nausea 60.0% (95%CI: 50.1%-69.1%, k=2, I^2^ = 0.0%), decreased appetite 50.0% (95%CI: 36.6%-63.3%, k=4, I^2^ = 68.4%), diarrhea 39.2% (95%CI: 31.8%-47.2%, I^2^ = 0.0%), and dysgeusia 32.0% (95%CI: 24.4%-40.7%, I^2^ = 0.0%). Constipation and vomiting occurred at rates of 26.3% and 21.6%, respectively. Among constitutional symptoms, fatigue was the most common with a pooled incidence of 42.4% (95%CI: 21.9%-66.0%, k=5, I^2^ = 89.3%, p<0.001), and asthenia was 29.6% (95%CI: 22.3%-38.2%, I^2^ = 0.0%). For cutaneous toxicities, maculopapular rash or rash occurred in 17.4% (95%CI: 8.8%-31.5%, I^2^ = 55.0%). Regarding hepatic toxicities, elevated aspartate aminotransferase and elevated alanine aminotransferase were 16.4% (95%CI: 10.9%-23.9%, I^2^ = 0.0%) and 14.6% (95%CI: 8.5%-24.0%, I^2^ = 0.0%), respectively. For neurologic toxicities, the pooled incidence of peripheral sensory neuropathy was 50.6%, but with very high between-study heterogeneity (95%CI: 9.1%-91.3%, I^2^ = 94.8%, p<0.001), warranting cautious interpretation. Endocrine toxicities included hypothyroidism (9.4%, 95%CI: 4.4%-19.1%) and hyperthyroidism (7.3%, 95%CI: 3.4%-14.8%), with adrenal insufficiency at 6.0% (95%CI: 2.7%-12.7%). Immune-related pneumonitis had a pooled incidence of 5.0% (95%CI: 2.1%-11.5%, I^2^ = 21.2%).

We performed an additional leave-one-out sensitivity analysis for grade ≥3 TRAEs, and the results have been added to the **Supplementary Materials** (**Supplementary Table 5**). The pooled incidence of grade ≥3 TRAEs remained within the range of 0.50 to 0.66 after sequential exclusion of individual studies, with overlapping 95% CIs across all estimates. No single study altered the overall conclusion, suggesting that the pooled estimate was robust. Notably, exclusion of Yung2019b reduced heterogeneity from approximately I²=82% to 33%, indicating that this study was a major contributor to the observed heterogeneity. For any-grade and grade ≥3 irAEs, leave-one-out sensitivity analyses were not performed because only two studies reported standardized pooled irAE counts, which was below the minimum number of studies generally required for such analyses (at least three studies).

#### 3.4.6 Immune-related adverse events

The pooled incidence rates of irAEs are detailed in **Table 3**. Across the three RCTs that included 1730 patients in the pembrolizumab group, the pooled incidence of any grade irAE was 31.1% (95%CI: 19.4%-45.8%, k=3, I^2^ = 96.9%, p<0.001), and that of grade 3 or higher irAE was 7.8% (95%CI: 5.8%-10.4%, I^2^ = 65.2%, p=0.056). The most common irAEs were hypothyroidism (21.4%, 95%CI: 10.9%-37.7%) and hyperthyroidism (6.4%, 95%CI: 4.8%-8.5%), followed by immune related pneumonitis (3.3%, 95%CI: 2.4%-4.4%), colitis (3.0%, 95%CI: 2.2%-4.2%), severe skin reactions (2.6%, 95%CI: 1.6%-4.3%), and hepatitis (1.4%, 95%CI: 0.9%-2.3%). Although rare, myocarditis (0.2%) and vasculitis (0.5%) are potentially fatal, and their clinical monitoring should not be overlooked.

**Table 3:** Pooled incidence of immune-related adverse events (irAEs) in patients receiving pembrolizumab-based regimens.

System/adverse event	No. of studies (k)	Events/total	Pooled rate (%)	95% CI	I² (%)	p (Q test)
Gastrointestinal						
Colitis	2	37/1226	3	2.2–4.2	0	0.423
Pancreatitis	2	9/1226	0.8	0.2–2.6	69.2	0.071
Skin						
Severe skin reactions	2	31/1226	2.6	1.6–4.3	52	0.149
Hepatic						
Hepatitis	2	17/1226	1.4	0.9–2.3	0	0.342
Endocrine						
Hypothyroidism	2	248/1226	21.4	10.9–37.7	96.9	<0.001
Hyperthyroidism	2	77/1226	6.4	4.8–8.5	40.5	0.195
Adrenal insufficiency	2	23/1226	2	0.9–4.4	75.4	0.044
Thyroiditis	2	11/1226	0.9	0.4–2.0	29.6	0.233
Hypophysitis	2	8/1226	0.7	0.2–2.0	55.2	0.135
Type1 diabetes	2	7/1226	0.6	0.3–1.2	0	0.684
Respiratory						
Pneumonitis	2	40/1226	3.3	2.4–4.4	0	0.838
Renal						
Nephritis	2	9/1226	0.8	0.4–1.7	30.2	0.231
Cardiovascular						
Vasculitis	2	5/1226	0.5	0.2–1.2	14	0.281
Myocarditis	2	2/1226	0.2	0.0–1.5	35.4	0.214
Musculoskeletal						
Myositis	2	2/1226	0.2	0.0–0.7	0	0.683
Overall_irAE						
AnyGrade irAE n	3	529/1730	31.1	19.4–45.8	96.9	<0.001
Grade35 irAE n	3	135/1730	7.8	5.8–10.4	65.2	0.056

The pooled incidence rates of irAEs in single-arm studies are presented in **Supplementary Table 6**. A total of 6 types of irAEs were reported in at least 2 studies and were included in the pooled analysis. Among endocrine system irAEs, hyperthyroidism (10.7%, 95%CI: 4.9%-21.9%; k=2, I^2^ = 19.4%) and hypothyroidism (7.1%, 95%CI: 2.7%-17.5%; k=2, I^2^ = 0.0%) were predominant. The pooled incidence of immune related pneumonitis was 8.3% (95%CI: 4.0%-16.5%; k=3, I^2^ = 0.0%), severe skin reactions 7.1% (95%CI: 2.7%-17.5%; k=2, I^2^ = 28.8%), colitis 4.8% (95%CI: 1.8%-12.0%; k=3, I^2^ = 0.0%), and infusion related reactions 3.6% (95%CI: 0.9%-13.2%; k=2, I^2^ = 0.0%). Between-study heterogeneity for each irAE was low (all I^2^≤ 28.8%), indicating relatively consistent reporting of irAEs across single-arm studies. Compared with the RCT component, the single-arm studies included a smaller number of studies and smaller sample sizes, limiting the types of irAEs that could be reported; nevertheless, the incidence rates of the reported irAEs were generally directionally consistent with the RCT data.

**Figure 8:**
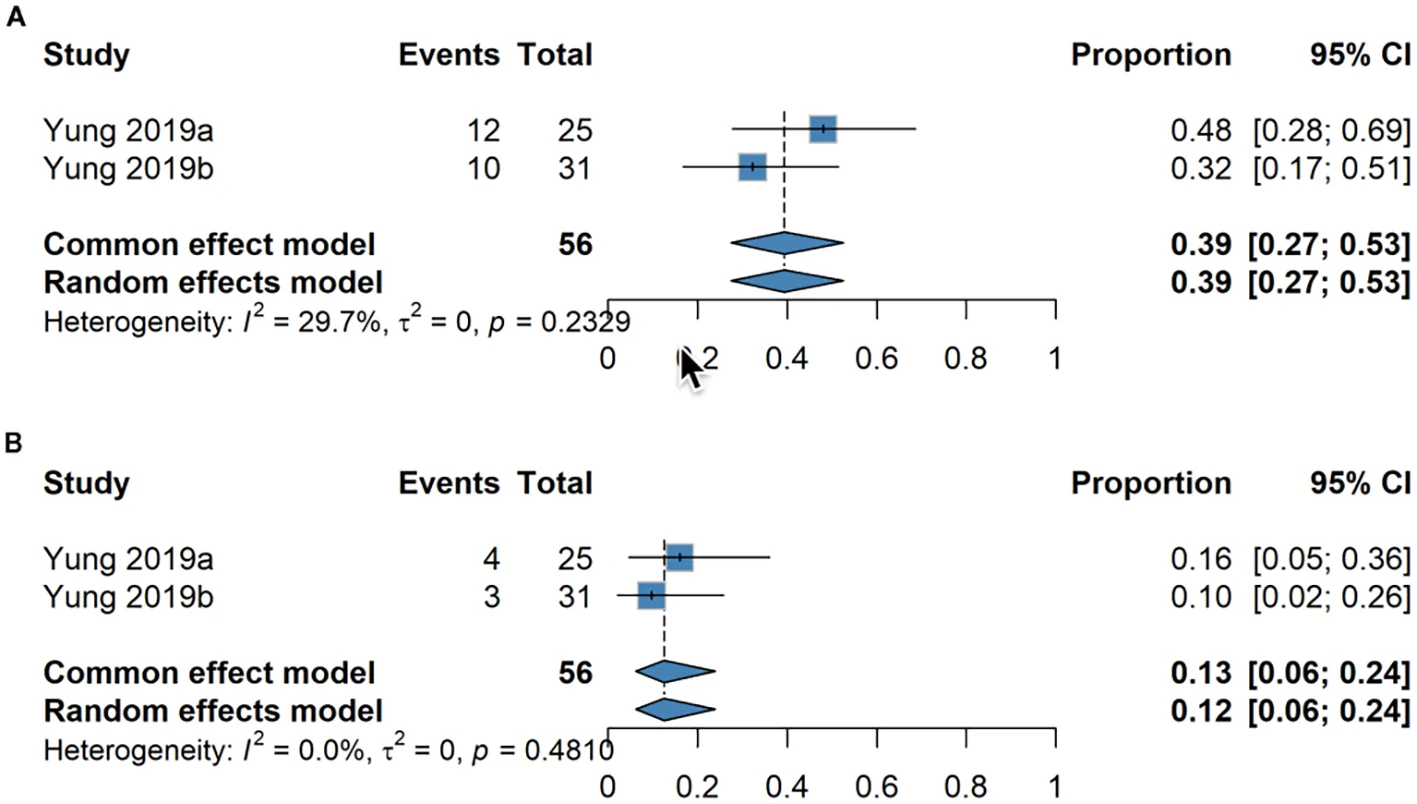
Pooled irAEs of single-arm studies. **(A)** Any-grade irAEs of single-arms; **(B)** Grade ≥3 irAEs of single-arms.

We performed standardized pooled analyses of irAEs reported in the included studies according to two severity categories: any-grade irAEs and grade ≥3 irAEs (**Figures 8A, B**). The pooled incidence of any-grade irAEs was 0.39 (95%CI: 0.27-0.53, I²=29.7%), whereas the pooled incidence of grade ≥3 irAEs was 0.12 (95%CI: 0.06-0.24, I²=0%).

#### 3.4.7 Subgroup analysis

**Figure 9:**
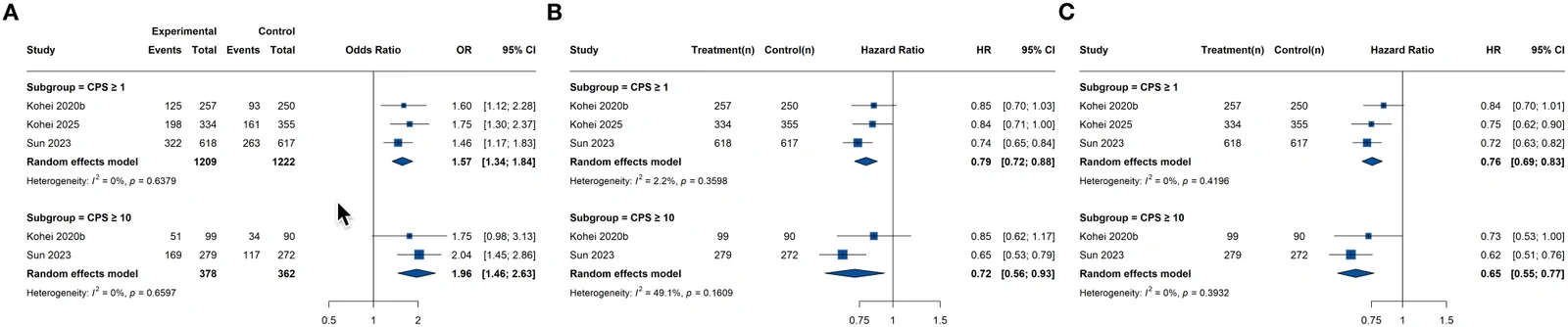
Incidence and subgroup analysis **(A)** Subgroup ORR of RCTs; **(B)** Subgroup OS of RCTs; **(C)** Subgroup PFS of RCTs.

Subgroup analysis stratified by PD-L1 CPS. For ORR, in the CPS at least 1 population, pembrolizumab plus chemotherapy significantly improved the ORR compared with the control group (odds ratio=1.57, 95%CI: 1.34-1.84; k=3, I^2^ = 0%). The benefit was more pronounced in the CPS at least 10 population (odds ratio=1.96, 95%CI: 1.46-2.63; k=2, I^2^ = 0%). For OS, the pooled HR was 0.79 (95%CI: 0.72-0.88; k=3, I^2^ = 2.2%) in the CPS at least 1 population, and 0.72 (95%CI: 0.56-0.93; k=2, I^2^ = 49.1%) in the CPS at least 10 population. For PFS, the pooled HR was 0.76 (95%CI: 0.69-0.83; k=3, I^2^ = 0%) in the CPS at least 1 population, and 0.65 (95%CI: 0.55-0.77; k=2, I^2^ = 0%) in the CPS at least 10 population. All three efficacy endpoints showed a consistent pattern: the magnitude of benefit was greater in the CPS at least 10 population than in the CPS at least 1 population, and between-study heterogeneity within each subgroup was low (**Figures 9A-C**). Similarly, the data showed that, among patients receiving pembrolizumab monotherapy, the ORR was 14.8%, with an OS HR of 0.91 (95%CI: 0.74-1.10) and a PFS HR of 1.66 (95%CI: 1.37-2.01) in the CPS≥1 subgroup. In the CPS≥10 subgroup, the ORR was 25%, with an OS HR of 0.69 (95%CI: 0.49-0.97) and a PFS HR of 1.10 (95%CI: 0.79-1.51). These findings suggest that, among patients with HER2-negative advanced GC or GEJC with CPS≥10, pembrolizumab monotherapy showed an OS benefit in the CPS≥10 subgroup (HR 0.69), but this was not accompanied by a PFS improvement (HR 1.10), highlighting the critical role of combination chemotherapy in disease control. To further explore whether treatment strategy influences the predictive performance of PD-L1 expression, we performed a *post hoc* assessment of the interaction between treatment modality (combination therapy versus monotherapy) and CPS subgroup based on the available RCT data. However, it should be noted that among the four RCTs included in this meta-analysis, only the Kohei 2020 study incorporated a pembrolizumab monotherapy arm. Therefore, a robust formal interaction test between treatment strategy and CPS status could not be performed at the meta-analytic level. The above findings should be interpreted as descriptive comparisons based on an individual study rather than definitive evidence of treatment-modality interaction. Nevertheless, the consistent direction of the observed efficacy trends supports the potential robustness of CPS≥10 as a predictive threshold across different treatment strategies.

Subgroup analysis of single-arm studies stratified by PD-L1 CPS. The pooled overall ORR was 0.23 (95%CI: 0.14-0.34, I^2^ = 52.9%). The CPS below 1 subgroup had the ORR at 0.12 (95%CI: 0.03-0.31), whereas the CPS at least 1 subgroup had an ORR of 0.3 (95%CI: 0.2-0.41, I^2^ = 42.5%), with a statistically significant difference across subgroups (p=0.0001), the CPS at least 10 subgroup had the ORR at 0.14 (95%CI: 0.04-0.33) (**Supplementary Figure 5**). The pooled mOS was 16.54 months (95%CI: 14.03-19.50, I^2^ = 0.0%), and no significant difference was observed among subgroups (p=0.54) (**Supplementary Figure 6**). The pooled mPFS was 10.08 months (95%CI: 6.75-15.05, I^2^ = 10.2%). The CPS at least 10 subgroup had the numerically highest mPFS at 8.12 months (95%CI: 5.49-12.02), but the difference across subgroups did not reach statistical significance (p=0.97). For the subgroup with CPS below 10, the Kensei 2022 study reported a value of 14.80 (95% CI: 8.80-24.89), whereas for the CPS at least 1 subgroup, the Ian 2020 study reported a value of 8.60 (95% CI: 2.87-25.80)(**Supplementary Figure 7**). It should be noted that the subgroup analyses of the single-arm studies were not adjusted for baseline covariates, such as ECOG performance status, prior treatment, or metastatic burden, all of which are known to interact with both PD-L1 expression and clinical prognosis. Within the ROBINS-I framework, the absence of adjustment for these potential confounders further increases the risk of confounding in these subgroup estimates. Therefore, the observed trends, such as the numerically longer mPFS in the CPS≥10 subgroup, should be regarded as exploratory findings and interpreted with caution until confirmed in adequately controlled RCTs.

To further explore the potential sources of high heterogeneity (I^2^>90%) in efficacy and safety outcomes, additional stratification analyses were performed by geographic region, median follow-up, and chemotherapy backbone. For single-arm studies, stratification by geographic region into Asia-only (k=2) and Global-Western (k=3) groups significantly reduced intra-subgroup heterogeneity (overall ORR I^2^ = 88.5%). The pooled ORR was significantly superior in the Asia-only subgroup compared with the Global-Western subgroup (76.0% *vs*. 35.6%,P=0.0002; **Supplementary Figure 8**). Similarly, the pooled mPFS was significantly prolonged in Asian studies (9.05 *vs*. 5.11, P = 0.0357; **Supplementary Figure 9**). Nevertheless, given that the Asian-only subgroup included only two single-arm studies, these statistically significant findings are hypothesis-generating rather than confirmatory, and should be interpreted with extreme caution due to the high risk of small-study effects and potential confounding. However, no statistically significant differences were observed between the two regions regarding pooled mOS (16.96 *vs*. 19.05, P = 0.5612; **Supplementary Figure 10**) or the incidence of Grade≧3 TRAES (68.8% *vs*. 46.7%,P=0.1371; **Supplementary Figure 11**).

#### 3.4.8 Sensitivity analysis

The leave-one-out sensitivity analysis for the RCT component showed that the OS results were robust. After sequentially omitting each study, the pooled HRs ranged from 0.811 to 0.870, and neither the direction of effect nor statistical significance was altered. The results for PFS and ORR were sensitive to individual studies. After removing Kohei 2020, the pooled HR for PFS was 0.767 (95%CI: 0.696-0.845, I^2^ = 0%), whereas after removing Kohei 2025 or Sun 2023, the HR increased to 0.943 and 0.960, respectively, with confidence intervals crossing the null and I^2^ rising to 95.6% and 92.9%. A similar pattern was observed for ORR. After removing Kohei 2020, the odds ratio was 1.57 (95%CI:1.31-1.88, I^2^ = 21.6%), but after removing Kohei 2025 or Sun 2023, the odds ratio decreased to 1.08 and 1.18, respectively, neither of which retained statistical significance. These findings indicate that Sun 2023 contributed substantially to the primary analyses of PFS and ORR, exerting strong weight on the pooled effect estimates as the study with the largest sample size. Our sensitivity analyses demonstrated that the statistical significance of PFS and ORR in the RCT cohort was highly dependent on the inclusion of several dominant trials, particularly those conducted by Sun et al. (32) and Kohei et al. (33). These findings indicate that the current evidence supporting the superiority of pembrolizumab combination therapy over monotherapy for these short term endpoints relies substantially on large scale studies with high statistical power. Excluding these key trials inevitably reduced the overall statistical power and increased the relative influence of smaller studies with baseline heterogeneity, thereby resulting in the loss of statistical significance.

For the single-arm study component, leave-one-out sensitivity analysis revealed that after sequentially excluding each study, the pooled incidence rate of ORR ranged from 45.6% to 61.2%, the pooled mOS ranged from 16.8 to 17.9 months (with I^2^=consistently 0%), and the pooled mPFS ranged from 5.81 to 7.69 months. Neither the direction nor the magnitude of effect for any endpoint was substantially altered, indicating that the primary analysis results for the single-arm studies were robust (**Supplementary Tables 7**, **8**). Although the sensitivity analysis of the single arm cohorts demonstrated remarkable consistency in absolute clinical outcomes, such as mOS and mPFS, with no observed heterogeneity (I²=0%), these findings should be interpreted with caution. Single arm studies inherently lack a concurrent head to head control group. Therefore, the high stability observed across these independent cohorts only supports the predictable and reproducible baseline efficacy of the combination regimen in different clinical settings. Methodologically, however, these findings cannot be used to directly estimate or infer the comparative advantage of combination therapy over monotherapy.

#### 3.4.9 Publication bias

**Figure 10:**
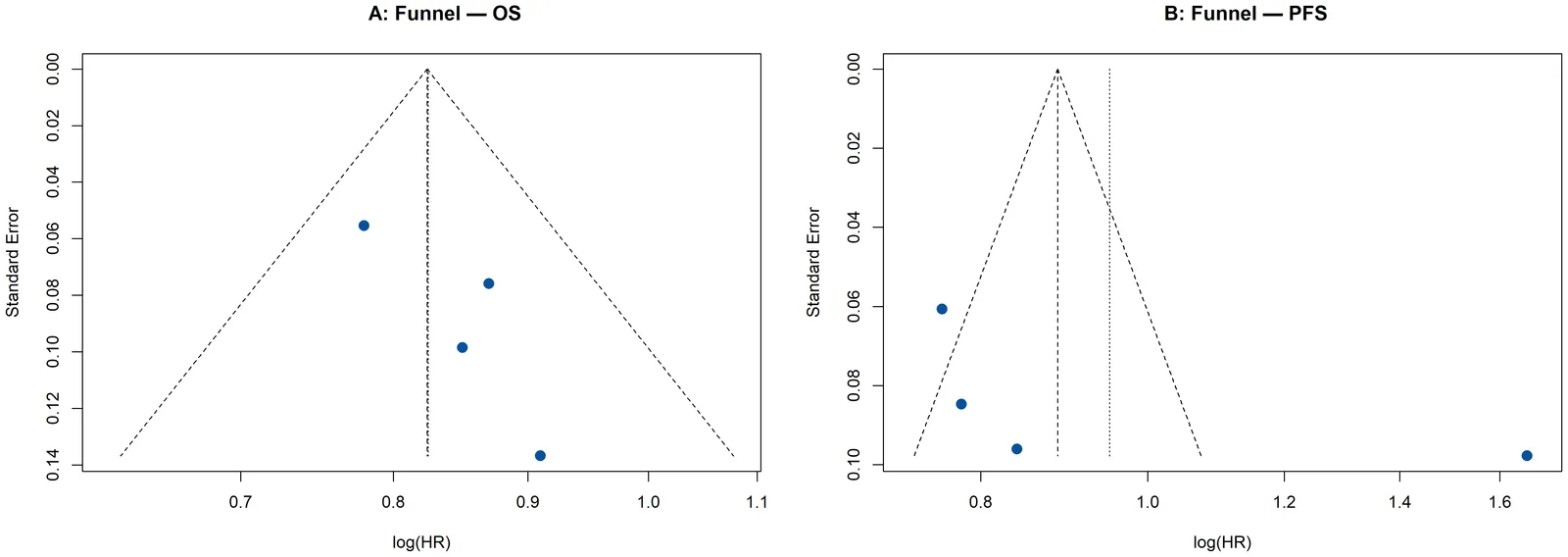
Publication bias assessment. **(A)** Funnel-OS; **(B)** Funnel-PFS.

Funnel plots for each outcome in the RCTs and single-arm studies are shown in **Figures 10A, B**, respectively. Visual inspection revealed that the funnel plots for OS and ORR in the RCTs were roughly symmetric, whereas one study in the funnel plot for PFS deviated from the funnel region. In the single-arm studies, the distribution of mOS was relatively symmetric, while the studies for ORR and mPFS were more widely scattered, likely reflecting clinical heterogeneity in treatment regimens and patient characteristics. The RCTs included three studies, and the single-arm studies included four to five studies, both fewer than ten. Following the recommendation of the Cochrane Handbook, the Egger test was not performed. The current evidence is insufficient to determine whether publication bias is present (**Supplementary Figures 12**, **13**).

## 4 Discussion

Pembrolizumab is a highly selective humanized IgG4 monoclonal antibody that binds specifically to PD-1 on the surface of T lymphocytes, thereby blocking its interaction with the ligands PD-L1 and PD-L2 (39). This blockade disrupts tumor-mediated immunosuppressive signaling and restores the antitumor effector functions of T cells. The agent has demonstrated substantial clinical utility across a diverse range of solid malignancies, and its investigation in the context of advanced GC has attracted considerable interest. However, previous studies evaluating pembrolizumab in HER2-negative advanced GC or GEJC have yielded inconsistent conclusions, particularly with respect to the comparative advantages of monotherapy versus combination chemotherapy, the differential treatment benefits across patient subsets stratified by PD-L1 expression, and the comprehensive characterization of the safety profile. Although previous meta-analyses have provided broad evaluations of immunotherapy in GC, a more detailed quantitative synthesis is still needed to clarify how different clinical study designs (RCTs versus exploratory single-arm studies) influence the interpretation and quality of evidence specifically in HER2-negative GC. By conducting stratified analyses that differentiated findings based on study design, therapeutic regimen and PD-L1 expression status, age, region, chemo backbone, and median follow-up, this investi1gation not only substantiates the therapeutic value of pembrolizumab but also elucidates the critical determinants that modulate its clinical activity. The findings presented herein provide a robust evidence base to inform precision-oriented clinical decision making and to guide the conceptualization of future investigative endeavors.

The findings of this meta-analysis demonstrate that pembrolizumab exhibits definitive and clinically meaningful antitumor activity in the management of HER2-negative advanced GC or GEJC. With respect to ORR, the aggregated data from RCTs indicate a trend toward improvement with pembrolizumab relative to conventional chemotherapy, although the overall effect size is markedly influenced by the specific treatment modality employed. Sensitivity analyses further delineate this relationship, revealing that pembrolizumab administered in conjunction with chemotherapy yields a significantly elevated response rate, and the corresponding odds ratio compared with the control arm attains statistical significance, with a high degree of consistency observed across the three major randomized trials. In contrast, the response rate associated with pembrolizumab monotherapy remains comparatively modest and does not demonstrate a discernible advantage over chemotherapy. This observation is corroborated by the pooled analysis of single-arm studies wherein the aggregate response rate in cohorts, receiving combination chemotherapy substantially exceeds that of the monotherapy cohorts, thereby identifying the therapeutic strategy as a principal contributor to the overall heterogeneity.

The superior clinical efficacy of pembrolizumab combined with chemotherapy is supported by a strong translational and tumor immunological rationale. Emerging evidence suggests that conventional first-line chemotherapeutic agents, including fluoropyrimidines and platinum compounds, function not only through direct cytotoxic effects but also as important modulators of the tumor immune microenvironment. First, chemotherapy can induce immunogenic cell death (ICD) in GC cells. This process promotes the release of damage-associated molecular patterns (DAMPs) from tumor cells, which facilitates the recruitment and maturation of dendritic cells (DCs), enhances antigen presentation, and subsequently activates CD8^+^ T cell-mediated adaptive antitumor immune responses (40). Second, chemotherapy may help overcome the complex immunosuppressive barriers within GC. Recent translational studies have demonstrated that conventional chemotherapy can remodel the tumor microenvironment by reducing the infiltration and immunosuppressive activity of Tregs and myeloid-derived suppressor cells (MDSCs), thereby alleviating local immune tolerance. These effects may synergize with pembrolizumab-mediated blockade of the PD-1/PD-L1 pathway and reversal of T-cell exhaustion, leading to enhanced antitumor immunity (41) Furthermore, chemotherapy-induced inflammatory responses within the tumor microenvironment, including interferon-γ (IFN-γ)-associated immune activation, may promote adaptive upregulation of PD-L1 and major histocompatibility complex class I (MHC-I) molecules on tumor cells. This mechanism may enhance immune cell infiltration and facilitate the transition of gastric cancer from an immunologically “cold” tumor phenotype toward a more “inflamed” or “hot” tumor phenotype, thereby providing a more favorable context for PD-1 blockade therapy (42). In contrast, pembrolizumab monotherapy lacks the additional effects of chemotherapy-induced immune remodeling and antigen release amplification. Therefore, its activity may be relatively limited in the highly heterogeneous stromal and immunosuppressive environment of GC, which may partially explain why clinical outcomes observed in single-agent cohorts were less favorable than those achieved with combination therapy in our analysis. Collectively, these multidimensional synergistic mechanisms provide a strong biological rationale supporting the use of pembrolizumab combined with chemotherapy as a first-line therapeutic strategy.

To further validate the clinical reliability of our findings, we performed a comprehensive comparison between the pooled results of the present meta analysis and those reported in the two landmark first line immunotherapy trials for HER2-negative advanced GC or GEJC, KEYNOTE-859 and CheckMate-649. Regarding OS, the pooled survival benefit observed in our study (HR = 0.82) was highly consistent with those reported in KEYNOTE-859 (HR = 0.78) and CheckMate-649 (HR = 0.80), providing strong evidence for the robust and generalizable survival benefit of pembrolizumab plus chemotherapy in patients with HER2-negative advanced GC or GEJC. With respect to the ORR, our pooled estimate (53%) was highly consistent with that reported in KEYNOTE-859 (51.3%), indicating stable short term antitumor efficacy. Although CheckMate-649 reported a slightly higher ORR (58%), this difference may be attributable to variations in chemotherapy backbones, particularly the greater use of oxaliplatin based regimens, or intrinsic differences between PD-1 inhibitors. Notably, the comparable OS benefits observed across these studies suggest that modest differences in early tumor response do not substantially influence the long term survival benefit. Furthermore, the pooled PFS benefit in our study (HR: 0.76–0.78) was also highly consistent with the findings of these landmark trials. Regarding safety, the pooled incidence of grade ≧3 TRAEs was 56.6%, which was numerically lower than that reported in KEYNOTE-859 (59.0%) and CheckMate-649 (59.1%). This modest difference may be attributable to the inclusion of studies with more diverse chemotherapy backbones and broader patient populations in the present meta analysis, resulting in a pooled estimate that is more representative of routine clinical practice. These findings further suggest that the combination regimen maintains a favorable and manageable safety profile when applied in a broader clinical setting.

Several recent systematic reviews and meta-analyses have evaluated pembrolizumab in GC, providing important background for our study. Mansoor et al. (43) and Yang et al. (44) reported significant survival benefits with pembrolizumab plus chemotherapy. These findings are consistent with our main results and collectively support the combination regimen. On this basis, we sought to refine the evidence for a more specific population. First, whereas previous meta-analyses (21) often pooled patients with different HER2 statuses, focused on HER2-positive cases (22), or mixed various PD-1/PD-L1 inhibitors (23), we restricted our analysis to HER2-negative disease, given that this subgroup accounts for over 85% of GC in Asian populations and lacks targeted therapies, allowing a more precise evaluation in this setting. Second, with regard to methodology, some prior reviews (21, 43, 44) combined RCTs and single-arm studies. Rather than merging these designs, we performed separate subgroup analyses, preserving the causal inference strength of RCTs while using single-arm data as complementary information for real-world or exploratory contexts. Third, we further stratified pembrolizumab monotherapy and pembrolizumab plus chemotherapy. Sensitivity analysis showed that, after excluding regimen-related confounding, the heterogeneity for ORR and PFS in the combination-chemotherapy subgroup (RCTs only) dropped to 0%, suggesting that differences in treatment regimen and study design mixture may be important sources of heterogeneity in previous pooled analyses. Our stratified approach helps to clarify the net benefit of combination chemotherapy. In addition, our search strategy had no geographic restrictions, and although the included studies were predominantly Asian, we also incorporated data from European and North American populations, aiming for robust conclusions from a broad evidence base. In summary, building on the conclusions of previous meta-analyses, our study provides more targeted evidence for first-line immunotherapy decisions in HER2-negative GC or GEJC through refined population definition and stratified analyses. In the rapidly evolving landscape of first-line immunotherapy for advanced GC, comparisons between pembrolizumab and other PD-1 inhibitors have important clinical implications. Although the present study was not designed as a network meta-analysis, a recent comprehensive network meta-analysis, including 6,294 patients from six RCTs, provided valuable comparative insights. This analysis suggested that sintilimab plus chemotherapy ranked highest for OS (SUCRA value: 85.2%), whereas nivolumab plus chemotherapy ranked highest for PFS (96.8%) and ORR (82.9%). Notably, all four PD-1 inhibitors (sintilimab, pembrolizumab, nivolumab, and tislelizumab) combined with chemotherapy significantly improved mOS and ORR compared with chemotherapy alone. Regarding safety, pembrolizumab monotherapy achieved the highest SUCRA value for grade ≥3 TRAEs, whereas tislelizumab and sintilimab combined with chemotherapy did not increase the overall incidence of adverse events. However, direct comparisons across trials remain challenging due to substantial heterogeneity in geographic populations (Asian versus global populations), chemotherapy backbones (S-1-based versus capecitabine-based regimens), and PD-L1 assessment methods. Therefore, although pembrolizumab remains a validated first-line treatment option, therapeutic selection among different PD-1 inhibitors should be individualized based on PD-L1 expression level, patient comorbidities, toxicity profiles, and regional drug accessibility.

With regard to survival outcomes, the results obtained in this study are encouraging. Pooled analyses of the RCTs reveal that pembrolizumab-based therapy yields statistically significant prolongation of both OS and PFS. Specifically, the pembrolizumab-containing regimen is associated with a meaningful reduction in the risk of death and the risk of disease progression. In a pattern consistent with the ORR findings, this survival advantage is driven primarily by the combination chemotherapy approach. In sensitivity analyses restricted to trials evaluating combination regimens, the reductions in the hazards of mortality and progression conferred by pembrolizumab plus chemotherapy remain statistically significant and interstudy heterogeneity is negligible, underscoring the robustness of the conclusion. Conversely, pembrolizumab monotherapy does not demonstrate a survival benefit relative to chemotherapy and even exhibits a trend toward inferior mPFS. The aggregated data from single-arm studies offer an alternative perspective with numerically appreciable mOS durations. However, variations are evident across different treatment cohorts. Given that single-arm studies are predominantly exploratory Phase II investigations characterized by limited sample sizes and potential selection biases, extrapolation of these findings necessitates cautious interpretation.

To further delineate the patient subset most likely to derive benefit, subgroup analyses were conducted based on PD-L1 CPS. The findings indicate that among the RCTs evaluating pembrolizumab in combination with chemotherapy, both the PD-L1 CPS of 1 or greater and CPS of 10 or greater populations attained meaningful improvements in ORR, OS and PFS. Notably, a consistent gradient pattern emerged across all efficacy endpoints wherein the magnitude of benefit observed in the CPS of 10 or greater subgroup substantially exceeded that documented in the CPS of 1 or greater subgroup. This observation solidifies the role of PD-L1 expression level as a pivotal predictive biomarker for pembrolizumab plus chemotherapy and suggests that in routine clinical practice, patients whose tumors exhibit high PD-L1 expression exemplified by a CPS of 10 or greater are more likely to experience profound and durable survival gains from this combination regimen. The value of PD-L1 CPS as a predictive biomarker for pembrolizumab efficacy has garnered broad recognition. A dedicated meta-analysis of immunotherapy in GC explicitly noted that employing CPS as a threshold provides a more reliable prediction of mortality risk reduction compared with the tumor proportion score in the context of PD-1 or PD-L1 inhibitor therapy (45). A recently presented updated analysis of the KEYNOTE 859 trial focusing on the Asian population further demonstrated that with extended median follow-up pembrolizumab plus chemotherapy continued to confer sustained improvements in OS, PFS and ORR among Asian patients with no emergent safety signals identified.The mOS was significantly prolonged to 17.3 months compared with 13.0 months in the placebo group (HR = 0.75, 95%CI: 0.62–0.91). Similarly, mPFS was significantly improved (8.4 *vs*. 5.8 months, HR = 0.72, 95%CI: 0.58–0.89), without the emergence of new safety signals (46). Moreover, findings from the KEYNOTE 811 study corroborated that PD-L1 CPS serves as a robust predictive biomarker for pembrolizumab efficacy in HER2-positive GC or GEJC. Among patients with PD-L1 CPS ≥1, the addition of pembrolizumab to trastuzumab-based anti-HER2 therapy and chemotherapy significantly prolonged mPFS to 10.9 months compared with 7.3 months in the control group (HR = 0.72, 95%CI: 0.60–0.87) and provided a significant OS benefit. In contrast, no clear clinical benefit was observed in the CPS <1 subgroup (mPFS: 9.5 *vs*. 9.5 months, HR = 1.03, 95%CI: 0.65–1.64) (47). Although that investigation specifically addressed a HER2-positive cohort the underlying biological rationale aligns with the observations derived from the HER2-negative population in the present study affirming a discernible enrichment effect between PD-L1 expression intensity and the clinical activity of pembrolizumab. Subgroup analyses of the single-arm studies, while constrained by limited sample sizes and incomplete data reporting did not yield a clearly defined dose response gradient however, the trends observed did not conflict with the evidence generated from the RCTs. Although the exploratory subgroup analyses yielded relatively robust consistent trends across key compliance strata, clinicians should still maintain an attitude of analytical prudence. When extrapolating our findings to specific patient populations, including the elderly, individuals with poor performance status (e.g., ECOG PS ≥2), or those presenting with unique metastatic patterns, it is essential to recognize that these sub-cohorts were either substantially underrepresented or lacked dedicated, prospective designs within the primary included clinical trials. Consequently, the efficacy and safety profiles of the evaluated regimens remains to be fully elucidated in these highly selective and vulnerable populations. Therefore, future dedicated clinical trials encompassing these specific cohorts are urgently needed.

Although this meta-analysis focused primarily on PD-L1 CPS, patient selection for immunotherapy in GC has entered the era of multi-biomarker assessment, with increasing attention being paid to emerging biomarkers such as MSI-H, TMB-H, and Epstein–Barr virus (EBV) positivity. Among these, MSI-H status is widely recognized as the most important predictive biomarker beyond PD-L1 CPS. In the prespecified subgroup analysis of the KEYNOTE-859 trial, patients with MSI-H tumors (n=74, 4.7% of the intention-to-treat population) achieved a substantially greater OS benefit from pembrolizumab plus chemotherapy than from chemotherapy alone (HR = 0.34), representing a 66% reduction in the risk of death and a markedly greater benefit than that observed in the overall study population (HR = 0.78) (32). Accordingly, the NCCN Gastric Cancer Guidelines (Version 1.2026) recommend universal MSI/MMR testing for all newly diagnosed patients with GC (48), and the CSCO Guidelines also list MSI/MMR testing as a Grade I recommendation (49). Therefore, in clinical practice, MSI-H status should take precedence over PD-L1 CPS when considering immunotherapy. Patients with MSI-H tumors should be considered for immunotherapy regardless of their PD-L1 CPS. TMB-H ≥10 mut/Mb may provide complementary predictive information, particularly for patients with low PD-L1 expression. In KEYNOTE-859, the TMB-H subgroup also demonstrated a significant OS benefit (HR = 0.61), and this benefit appeared to be independent of PD-L1 CPS expression (32). The CSCO Guidelines recognize TMB-H (≥10 mut/Mb) as a reference biomarker for immune checkpoint inhibitor treatment (Grade III recommendation) (49). Combined assessment of TMB and PD-L1 CPS may further optimize patient selection by identifying patients with low PD-L1 expression but high TMB who are likely to benefit from immunotherapy while reducing unnecessary treatment in patients with high PD-L1 expression but low TMB. In addition, the predictive value of TMB for immunotherapy efficacy has been validated in patients with advanced GC (50). EBV-positive GC, accounting for approximately 2%-10% of cases, represents another highly immunogenic molecular subtype. A single-center retrospective study including 293 patients treated with first-line nivolumab plus chemotherapy reported an EBV-positive rate of 6.1% (18/293), and multivariable analysis identified EBV positivity combined with PD-L1 CPS ≥5 as an independent predictor of PFS (51). Furthermore, an international multicenter retrospective analysis involving 91 patients with metastatic EBV-positive GC across nine centers reported an ORR of 49% and a median OS of 38 months with first-line immunotherapy plus chemotherapy (52). However, independent analyses of the EBV-positive subgroup have not yet been reported in KEYNOTE-859, and neither the NCCN nor the CSCO guidelines currently recommend routine EBV testing as part of the standard biomarker panel for immunotherapy decision-making.

This meta-analysis undertook a systematic synthesis of TRAEs and irAEs associated with pembrolizumab-based therapy. Within the RCTs the incidence of TRAEs of any grade was high in both the pembrolizumab arm and the control arm, and no statistically meaningful difference was observed between the two groups. However stratification according to therapeutic regimen revealed that the combination of pembrolizumab with chemotherapy was associated with a significantly elevated risk of grade 3 or higher severe adverse events as well as an increased likelihood of treatment discontinuation due to toxicity with the corresponding odds ratio attaining statistical significance. This observation underscores the clinical imperative that although the addition of immunotherapy to chemotherapy confers meaningful survival advantages, this benefit is accompanied by an incremental toxicity necessitating intensified patient surveillance and proactive management throughout the treatment course.

Through pooled analysis of specific adverse events, this investigation delineates the safety landscape of pembrolizumab-containing regimens. The most frequently encountered toxicities were concentrated within the hematologic system, for instance decreased neutrophil count and anemia, the gastrointestinal system, including nausea diarrhea and diminished appetite and constitutional symptoms such as fatigue. These adverse events overlap substantially with the well established toxicity profiles of the concomitant chemotherapeutic agents thereby confirming an additive toxic effect inherent to the combination approach. It is important to emphasize that irAEs constitute a distinct category of toxicity uniquely attributable to immunotherapy. The present study observed that any grade irAEs occurred relatively frequently among patients receiving pembrolizumab. Endocrinopathies specifically hypothyroidism and hyperthyroidism represented the most common manifestations followed by immune mediated pneumonitis colitis and hepatitis. Although the majority of irAEs are mild to moderate in severity and generally manageable rare instances of severe or potentially fatal events including myocarditis and adrenal insufficiency may arise.

With the expanding integration of immune checkpoint inhibitors into the therapeutic armamentarium for GC the management of irAEs has emerged as a central clinical priority. In a review published in the European Journal of Medical Research Yin and colleagues (53) comprehensively summarized potential biomarkers and mechanistic pathways governing irAEs in GC and emphasized that the majority of such events can be effectively controlled through early recognition and guideline-directed therapeutic interventions. Across esophageal squamous cell carcinoma and GC or GEJC the immune-related toxicity spectra of various PD-1 inhibitors, including nivolumab, pembrolizumab and tislelizumab, appear broadly comparable with thyroid dysfunction and other endocrine disorders being commonly encountered whereas the incidence of grade 3 or higher irAEs remains relatively low and clinically manageable (54). These consensus observations align with the incidence patterns and organ system distribution of irAEs documented in the present meta-analysis thereby providing additional support for the characterization of pembrolizumab plus chemotherapy as a regimen with a manageable safety profile in routine clinical practice. Early detection accurate grading and timely institution of appropriate interventions, including the administration of corticosteroids as well as temporary withholding or permanent cessation of pembrolizumab are critical measures to safeguard patient wellbeing and to optimize the therapeutic ratio. The pooled adverse event data derived from single-arm studies exhibit trends that are broadly consistent with those observed in the RCTs further enriching the available safety information pertaining to pembrolizumab in both real-world settings and exploratory clinical research contexts.

The present investigation encountered substantial heterogeneity in select pooled analyses, most notably within the assessments of overall treatment efficacy. Through comprehensive subgroup and sensitivity analyses, the principal sources underlying this heterogeneity were successfully identified and characterized. The choice of therapeutic strategy namely combination chemotherapy versus monotherapy, emerged as the predominant driver of observed variability. Incorporation of studies evaluating pembrolizumab monotherapy into the primary analyses not only introduced directional discordance in effect estimates but also markedly amplified statistical heterogeneity. When the analytic scope was restricted to combination chemotherapy regimens interstudy consistency improved considerably for both efficacy and safety outcomes. Moreover, the leave-one-out sensitivity analyses conducted within this framework demonstrated that the pooled estimate for OS, a pivotal endpoint remained robust and was not unduly influenced by any single trial. In contrast for PFS and ORR the largest registrational study exerted a substantial weighting on the aggregated results. This phenomenon appropriately reflects the inherent contribution of high-quality-large-scale investigations within meta-analysis methodology yet simultaneously underscores the necessity for circumspect interpretation of these specific endpoints with due consideration of potential influences from individual studies. Sensitivity analyses confined to the single-arm studies indicated that the respective pooled estimates exhibited satisfactory stability.

Although our primary analyses demonstrated high consistency across combination trials, several potential sources of residual heterogeneity warrant consideration. First, heterogeneity in backbone chemotherapy regimens (e.g., S-1-based *vs*. capecitabine-based regimens) and geographic distribution (Asian *vs*. Western populations) influenced efficacy and toxicity estimates, as demonstrated in our subgroup analyses. Second, variations in PD-L1 detection assays (e.g., 22C3 *vs*. 28–8 pharmDx) and evaluation platforms across clinical trials may have introduced minor measurement heterogeneity that could not be fully adjusted for in aggregate-data pooling. Third, minor discrepancies in baseline disease characteristics, such as previous gastrectomy status and specific histological subtypes, might also contribute to subtle variances in long-term survival outcomes. Future studies utilizing IPD are encouraged to perform pooled interaction analyses to clarify these underlying effect modifiers.

### 4.1 Analysis of strengths and limitations

First, this study strictly focused on the HER2-negative clinical setting, providing tailored evidence for this large and clinically vital patient population. Second, methodologically, we performed refined stratified analyses based on study design rather than pooling data indiscriminately. This allowed RCTs to serve as the primary basis for causal inference while evaluating single-arm studies independently as exploratory evidence, enhancing both methodological transparency and clinical applicability. Third, we conducted a granular secondary stratification to clearly differentiate pembrolizumab monotherapy from combination chemotherapy. Finally, this study implemented a comprehensive search strategy with no geographic restrictions. Although the included studies were predominantly in Asian populations, the inclusion of European and North American cohorts ensures the systematic nature and broader generalizability of the evidence base.

However, this study also has several limitations. First, substantial heterogeneity was observed across analyses, which remained partially unexplained despite subgroup exploration. Consequently, the pooled findings should be interpreted with caution and regarded as hypothesis-generating rather than confirmatory. Second, the limited number and quality of included studies introduced significant uncertainty into the OS and PFS estimates. These survival benefits lack definitive evidence and their clinical implications must be viewed as exploratory. Third, as noted in the sensitivity analysis, the single-arm estimates are subject to confounding and should be interpreted exploratorily. Fourth, the PD-L1 CPS subgroup analysis (CPS≧1 and CPS≧10) was severely constrained by overlapping data reporting. The lack of mutually exclusive strata precluded formal interaction testing, rendering the observed gradient effect purely descriptive. Fifth, the predominant inclusion of Asian populations may limit the generalizability of the findings to Western patients. Due to potential regional variations in clinical characteristics and treatment backbones, caution is required when extrapolating these results to Western subgroups.

### 4.2 Implications for clinical practice and future research

Based on the findings derived from the present investigation, the following considerations and recommendations are proposed for contemporary clinical practice and the direction of future research endeavors. (1) Optimization of therapeutic decision making. For patients diagnosed with HER2-negative advanced GC or GEJC, pembrolizumab administered in conjunction with chemotherapy should be regarded as a key standard first line treatment option, particularly for those subpopulations exhibiting a PD-L1 combined positive score of 1 or greater and most notably those with a score of 10 or greater. When electing to prescribe this regimen, clinicians are advised to thoroughly counsel patients regarding both the significant prolongation of survival and the attendant potential for incremental toxicity, and to establish a comprehensive prospective management plan for the surveillance and mitigation of adverse events. (2) To optimize the benefit-risk balance and avoid overtreatment, routine chemoimmunotherapy should be reconsidered for patients with low PD-L1 CPS, as clinical trials show increased grade ≥3 toxicity without a statistically significant survival benefit in this subgroup. Instead, treatment decisions should be strictly stratified by PD-L1 thresholds, while integrating complementary biomarkers like MSI-H and TMB-H to refine patient selection and spare non-responders from unnecessary high-grade toxicities. (3) Reinforcement of safety management protocols. In light of the elevated incidence of grade 3 or higher adverse events associated with the combination regimen, implementation of a multidisciplinary collaborative framework for monitoring and management is warranted. Surveillance efforts should concentrate on hematologic toxicities, gastrointestinal reactions, and the characteristic spectrum of irAEs. Upon the emergence of an irAE, management should be conducted in strict accordance with established international guidelines, including the judicious administration of corticosteroids and other immunosuppressive agents and the graded application of treatment interruption or permanent discontinuation, with the overarching objective of containing toxicity within acceptable limits while preserving therapeutic efficacy. (4) Direction of future investigational priorities. Future research initiatives should be directed toward several key areas. First, prospective head-to-head comparisons of different PD-1/PD-L1 inhibitors combined with chemotherapy are warranted. Current clinical trials have mainly evaluated individual ICIs in combination with chemotherapy versus chemotherapy alone, and existing systematic analyses have primarily summarized the efficacy and safety of different PD-1/PD-L1 inhibitor-based regimens without providing a clear classification or direct comparison among specific agents. Second, efforts should be intensified to identify and validate more precise predictive biomarkers beyond PD-L1 CPS, thereby enabling further enrichment of the responder population and sparing individuals unlikely to benefit from futile treatment exposure. Third, real-world studies are required to corroborate the findings reported herein, particularly through the assessment of long-term efficacy and safety profiles of pembrolizumab plus chemotherapy across diverse ethnic groups and patient subsets characterized by varying clinical attributes. Future meta-analyses based on IPD are essential to validate the optimal CPS cut-off values. Fourth, further accumulation of real-world evidence regarding long-term, chronic, and delayed irAEs is needed. The follow-up duration of current RCTs is insufficient to fully characterize the long-term safety profile of ICIs, particularly delayed toxicities with potential cumulative effects, such as endocrine dysfunction and cardiovascular complications. Large-scale registry-based real-world studies are urgently needed to clarify the incidence, risk factors, and impact of these adverse events on quality of life. Such evidence will be particularly important for patients who achieve long-term survival benefits from immunotherapy and will help guide individualized treatment strategies in clinical practice.

## 5 Conclusion

In summary, this systematic review and meta-analysis provides robust evidence drawn from the medical literature affirming that the combination of pembrolizumab and chemotherapy confers statistically significant and clinically meaningful survival advantages in the treatment of HER2-negative advanced GC or GEJC. These advantages encompass notable improvements in ORR as well as extensions in OS and PFS. The level of PD-L1 expression, particularly a CPS of 10 or greater, emerges as a pivotal biomarker for identifying individuals most likely to experience superior efficacy from the combination regimen. It must be acknowledged however that these survival gains are accompanied by an elevated risk of treatment related toxicities including an increased incidence of grade 3 or higher adverse events and irAEs. Nevertheless the overall safety profile remains within a spectrum that is both predictable and clinically manageable. The findings presented herein support the adoption of pembrolizumab plus chemotherapy as a standard first-line therapeutic strategy for patients with HER2-negative advanced GC or GEJC especially among those whose tumors exhibit PD-L1 positivity. Looking ahead the therapeutic horizon for pembrolizumab in this disease context appears increasingly promising. Through the implementation of more refined patient selection methodologies the optimization of toxicity surveillance and management protocols, and the execution of further large-scale prospective clinical investigations, it is anticipated that pembrolizumab will continue to foster more durable survival outcomes and contribute to an enhanced quality of life for affected individuals.

## Data Availability

The original contributions presented in the study are included in the article/**Supplementary Material**. Further inquiries can be directed to the corresponding author.
