# Supplementary material for: The efficacy and safety of pembrolizumab in the treatment of HER2-negative advanced gastric or gastroesophageal junction cancer: a systematic review and meta-analysis of emerging clinical data
Source: Front Immunol. 2026 Aug 19;17:1862751. doi: 10.3389/fimmu.2026.1862751 (PMC13534021; doi:10.3389/fimmu.2026.1862751)
Supplement: Supplementary file 1 [file DataSheet1.zip › Supplementary table/Supplementary Table S4_Common_trAEs _Single_arm.docx]

## 1.1. Supplementary Table S2. Pooled incidence of common treatment-related adverse events in single-arm studies of pembrolizumab (pooled rate ≥5%)

| System / Adverse event | No. of studies (k) | Events / Total | Pooled rate (%) | 95% CI | I² (%) | p (Q test) |
| --- | --- | --- | --- | --- | --- | --- |
| **Hematologic** |  |  |  |  |  |  |
| Decreased neutrophil count | 3 | 83 / 125 | 72.3 | 47.6–88.2 | 89.4 | <0.001 |
| Decreased platelet count | 3 | 48 / 125 | 36.4 | 22.5–53.0 | 78.9 | 0.009 |
| Anemia | 3 | 29 / 125 | 23.0 | 15.4–32.8 | 44.8 | 0.163 |
| Decreased WBC count | 3 | 20 / 125 | 16.0 | 10.6–23.5 | 0.0 | 0.671 |
| **Gastrointestinal** |  |  |  |  |  |  |
| Nausea | 2 | 60 / 100 | 60.0 | 50.1–69.1 | 0.0 | 0.870 |
| Decreased appetite | 4 | 80 / 153 | 50.0 | 36.6–63.3 | 68.4 | 0.024 |
| Diarrhea | 4 | 60 / 153 | 39.2 | 31.8–47.2 | 0.0 | 0.827 |
| Dysgeusia | 3 | 40 / 125 | 32.0 | 24.4–40.7 | 0.0 | 0.784 |
| Constipation | 4 | 43 / 153 | 26.3 | 15.8–40.5 | 75.4 | 0.007 |
| Vomiting | 4 | 33 / 153 | 21.6 | 15.8–28.8 | 0.0 | 0.650 |
| Hiccups | 3 | 14 / 125 | 11.1 | 5.1–22.1 | 58.2 | 0.091 |
| Colitis | 3 | 7 / 131 | 5.3 | 2.2–12.1 | 18.8 | 0.292 |
| **Skin** |  |  |  |  |  |  |
| Maculopapular rash or rash | 4 | 32 / 156 | 17.4 | 8.8–31.5 | 55.0 | 0.083 |
| Skin hyperpigmentation | 2 | 9 / 100 | 9.0 | 4.8–16.4 | 37.8 | 0.205 |
| **Constitutional** |  |  |  |  |  |  |
| Fatigue | 5 | 72 / 184 | 42.4 | 21.9–66.0 | 89.3 | <0.001 |
| Malaise | 3 | 37 / 125 | 29.6 | 22.3–38.2 | 0.0 | 0.384 |
| Edema peripheral | 3 | 17 / 128 | 13.4 | 6.6–25.4 | 70.4 | 0.034 |
| Pyrexia | 2 | 10 / 79 | 12.7 | 7.0–21.9 | 0.0 | 0.905 |
| **Hepatic** |  |  |  |  |  |  |
| Aspartate aminotransferase increased | 3 | 21 / 128 | 16.4 | 10.9–23.9 | 0.0 | 0.955 |
| Alanine aminotransferase increased | 2 | 12 / 82 | 14.6 | 8.5–24.0 | 0.0 | 0.554 |
| **Endocrine** |  |  |  |  |  |  |
| Hypothyroidism | 2 | 8 / 85 | 9.4 | 4.4–19.1 | 57.9 | 0.123 |
| Hyperthyroidism | 3 | 8 / 110 | 7.3 | 3.4–14.8 | 41.0 | 0.183 |
| Adrenal insufficiency | 2 | 6 / 100 | 6.0 | 2.7–12.7 | 0.0 | 0.840 |
| **Respiratory** |  |  |  |  |  |  |
| Pneumonitis | 2 | 5 / 100 | 5.0 | 2.1–11.5 | 21.2 | 0.260 |
| **Neurologic** |  |  |  |  |  |  |
| Peripheral sensory neuropathy | 3 | 67 / 125 | 50.6 | 9.1–91.3 | 94.8 | <0.001 |
| **Renal** |  |  |  |  |  |  |
| Increased blood creatinine level | 3 | 10 / 125 | 7.6 | 3.1–17.3 | 45.2 | 0.161 |

Abbreviations: CI, confidence interval; k, number of studies contributing data; Q, Cochran’s Q test for heterogeneity. Only adverse events with pooled incidence ≥5% are shown; complete data are available in Supplementary materials.
