# Supplementary material for: The efficacy and safety of pembrolizumab in the treatment of HER2-negative advanced gastric or gastroesophageal junction cancer: a systematic review and meta-analysis of emerging clinical data
Source: Front Immunol. 2026 Aug 19;17:1862751. doi: 10.3389/fimmu.2026.1862751 (PMC13534021; doi:10.3389/fimmu.2026.1862751)
Supplement: Supplementary file 1 [file DataSheet1.zip › Supplementary table/Supplenebtary Table S6_irAEs_Single_arm.docx]

## 1.1. Supplementary Table S4. Pooled incidence of immune-related adverse events in single-arm studies of pembrolizumab

| System / Adverse event | No. of studies (k) | Events / Total | Pooled rate (%) | 95% CI | I² (%) | p (Q test) |
| --- | --- | --- | --- | --- | --- | --- |
| **Endocrine** |  |  |  |  |  |  |
| Hyperthyroidism | 2 | 6 / 56 | 10.7 | 4.9–21.9 | 19.4 | 0.265 |
| Hypothyroidism | 2 | 4 / 56 | 7.1 | 2.7–17.5 | 0.0 | 0.823 |
| **Gastrointestinal** |  |  |  |  |  |  |
| Colitis | 3 | 4 / 84 | 4.8 | 1.8–12.0 | 0.0 | 0.770 |
| **Respiratory** |  |  |  |  |  |  |
| Pneumonitis | 3 | 7 / 84 | 8.3 | 4.0–16.5 | 0.0 | 0.496 |
| **Skin** |  |  |  |  |  |  |
| Severe skin reactions | 2 | 4 / 56 | 7.1 | 2.7–17.5 | 28.8 | 0.236 |
| **Other** |  |  |  |  |  |  |
| Infusion-related reaction | 2 | 2 / 56 | 3.6 | 0.9–13.2 | 0.0 | 0.877 |

Abbreviations: CI, confidence interval; k, number of studies contributing data; Q, Cochran’s Q test for heterogeneity. Only adverse events with pooled incidence ≥0% are shown; complete data are available in Supplementary materials.
