# Supplementary material for: The efficacy and safety of pembrolizumab in the treatment of HER2-negative advanced gastric or gastroesophageal junction cancer: a systematic review and meta-analysis of emerging clinical data
Source: Front Immunol. 2026 Aug 19;17:1862751. doi: 10.3389/fimmu.2026.1862751 (PMC13534021; doi:10.3389/fimmu.2026.1862751)
Supplement: Supplementary file 1 [file DataSheet1.zip › Supplementary table/Supplementary Table S7 _LOO_RCT.docx]

## 1.1. Supplementary Table S5. Leave-one-out sensitivity analysis (RCTs)

| Outcome | Excluded study | Pooled estimate (95% CI) | I² |
| --- | --- | --- | --- |
| OS | Kohei 2020 | 0.814 (0.733–0.903) | 25.9% |
| OS | Kohei 2025 | 0.811 (0.732–0.899) | 20.6% |
| OS | Sun 2023 | 0.870 (0.781–0.969) | 0.0% |
| PFS | Kohei 2020 | 0.767 (0.696–0.845) | 0.0% |
| PFS | Kohei 2025 | 0.943 (0.616–1.444) | 95.6% |
| PFS | Sun 2023 | 0.960 (0.643–1.432) | 92.9% |
| ORR | Kohei 2020 | 1.568 (1.306–1.883) | 21.6% |
| ORR | Kohei 2025 | 1.084 (0.593–1.981) | 90.4% |
| ORR | Sun 2023 | 1.183 (0.536–2.610) | 93.2% |
