# Supplementary material for: The efficacy and safety of pembrolizumab in the treatment of HER2-negative advanced gastric or gastroesophageal junction cancer: a systematic review and meta-analysis of emerging clinical data
Source: Front Immunol. 2026 Aug 19;17:1862751. doi: 10.3389/fimmu.2026.1862751 (PMC13534021; doi:10.3389/fimmu.2026.1862751)
Supplement: Supplementary file 1 [file DataSheet1.zip › Supplementary table/Supplementary Table S3 output_TableS1_All_trAEs.docx]

## 1.1. Supplementary Table S1. Pooled incidence of all reported treatment-related adverse events in patients receiving pembrolizumab-based regimens

| System / Adverse event | No. of studies (k) | Events / Total | Pooled rate (%) | 95% CI | I² (%) | p (Q test) |
| --- | --- | --- | --- | --- | --- | --- |
| **Hematologic** |  |  |  |  |  |  |
| Neutrophil count decreased | 3 | 454 / 1730 | 24.9 | 10.3–49.0 | 98.5 | <0.001 |
| Anemia | 3 | 447 / 1730 | 24.7 | 19.0–31.4 | 90.1 | <0.001 |
| Neutropenia | 2 | 230 / 1289 | 17.8 | 15.8–20.0 | 0.0 | 0.774 |
| Decreased platelet count | 3 | 338 / 1730 | 15.7 | 5.2–38.9 | 97.4 | <0.001 |
| WBC decreased | 3 | 235 / 1730 | 12.7 | 5.9–25.2 | 96.2 | <0.001 |
| Thrombocytopenia | 2 | 110 / 1289 | 7.7 | 3.9–14.6 | 90.3 | 0.001 |
| **Gastrointestinal** |  |  |  |  |  |  |
| Nausea | 3 | 650 / 1730 | 36.9 | 30.6–43.6 | 87.2 | <0.001 |
| Diarrhea | 3 | 500 / 1730 | 27.6 | 15.8–43.7 | 96.8 | <0.001 |
| Decreased appetite | 3 | 392 / 1730 | 22.9 | 17.4–29.6 | 88.1 | <0.001 |
| Vomiting | 3 | 369 / 1730 | 19.7 | 13.5–27.9 | 93.8 | <0.001 |
| Stomatitis | 2 | 99 / 945 | 10.2 | 4.8–20.2 | 92.7 | <0.001 |
| Mucosal inflammation | 2 | 94 / 945 | 10.0 | 7.4–13.4 | 58.3 | 0.121 |
| Constipation | 2 | 68 / 945 | 7.1 | 4.1–12.1 | 81.3 | 0.021 |
| Dysgeusia | 2 | 49 / 945 | 5.2 | 4.0–6.8 | 0.0 | 0.531 |
| **Skin** |  |  |  |  |  |  |
| PPE syndrome | 3 | 343 / 1730 | 18.6 | 11.9–27.8 | 93.2 | <0.001 |
| Rash | 2 | 87 / 945 | 9.2 | 7.5–11.3 | 0.0 | 0.444 |
| Pruritus | 2 | 80 / 945 | 8.5 | 6.8–10.7 | 16.0 | 0.275 |
| **Constitutional** |  |  |  |  |  |  |
| Fatigue | 3 | 375 / 1730 | 21.8 | 19.6–24.1 | 17.2 | 0.299 |
| Asthenia | 3 | 178 / 1730 | 9.9 | 6.8–14.1 | 81.0 | 0.005 |
| Weight decreased | 2 | 95 / 945 | 9.1 | 2.8–26.1 | 96.4 | <0.001 |
| **Hepatic** |  |  |  |  |  |  |
| AST increased | 2 | 220 / 1226 | 17.9 | 15.9–20.2 | 0.0 | 0.772 |
| ALT increased | 2 | 171 / 1226 | 14.2 | 11.5–17.3 | 52.8 | 0.145 |
| Bilirubin increased | 2 | 113 / 1226 | 9.2 | 7.4–11.3 | 25.6 | 0.246 |
| **Endocrine** |  |  |  |  |  |  |
| Hypothyroidism | 3 | 273 / 1730 | 15.3 | 7.7–28.2 | 96.7 | <0.001 |
| **Neurologic** |  |  |  |  |  |  |
| Peripheral neuropathy | 3 | 245 / 1730 | 11.9 | 5.3–24.6 | 95.5 | <0.001 |
| Peripheral sensory neuropathy | 3 | 224 / 1730 | 11.4 | 6.5–19.5 | 93.5 | <0.001 |
| **Metabolism** |  |  |  |  |  |  |
| Hypokalemia | 2 | 43 / 945 | 4.5 | 2.3–8.5 | 78.0 | 0.033 |

Abbreviations: CI, confidence interval; k, number of studies contributing data; Q, Cochran's Q test for heterogeneity. Only adverse events with pooled incidence ≥0% are shown; complete data are available in Supplementary Table S1.
