# Supplementary material for: The efficacy and safety of pembrolizumab in the treatment of HER2-negative advanced gastric or gastroesophageal junction cancer: a systematic review and meta-analysis of emerging clinical data
Source: Front Immunol. 2026 Aug 19;17:1862751. doi: 10.3389/fimmu.2026.1862751 (PMC13534021; doi:10.3389/fimmu.2026.1862751)
Supplement: Supplementary file 1 [file DataSheet1.zip › Supplementary table/Supplementary Table S8 _LOO_Single_arm.docx]

## 1.1. Supplementary Table S6. Leave-one-out sensitivity analysis (single-arm studies)

| Outcome | Excluded study | Pooled estimate (95% CI) | I² |
| --- | --- | --- | --- |
| ORR | Yung 2019a | 0.519 (0.255–0.772) | 91.4% |
| ORR | Yung 2019b | 0.612 (0.383–0.800) | 86.1% |
| ORR | Akihito 2020 | 0.481 (0.241–0.730) | 89.4% |
| ORR | Kensei 2022 | 0.456 (0.251–0.677) | 87.4% |
| ORR | Ian 2020 | 0.612 (0.386–0.799) | 86.7% |
| mOS | Yung 2019b | 16.830 (14.300–19.810) | 0.0% |
| mOS | Akihito 2020 | 17.900 (14.220–22.530) | 0.0% |
| mOS | Kensei 2022 | 17.400 (14.620–20.710) | 0.0% |
| mOS | Ian 2020 | 17.460 (14.960–20.370) | 0.0% |
| mPFS | Yung 2019a | 6.400 (3.930–10.440) | 72.8% |
| mPFS | Yung 2019b | 7.690 (6.120–9.660) | 6.3% |
| mPFS | Akihito 2020 | 5.810 (3.950–8.560) | 55.0% |
| mPFS | Kensei 2022 | 6.090 (3.910–9.490) | 72.1% |
| mPFS | Ian 2020 | 6.630 (4.380–10.040) | 72.7% |
