# Supplementary material for: The efficacy and safety of pembrolizumab in the treatment of HER2-negative advanced gastric or gastroesophageal junction cancer: a systematic review and meta-analysis of emerging clinical data
Source: Front Immunol. 2026 Aug 19;17:1862751. doi: 10.3389/fimmu.2026.1862751 (PMC13534021; doi:10.3389/fimmu.2026.1862751)
Supplement: Supplementary file 1 [file DataSheet1.zip › Supplementary table/Supplementary Table S1-Retrieval strategy.docx]

**Supplementary Table S1. Retrieval strategy**

| **number** | **Keywords of the research** |
| --- | --- |
| a. **Retrieval strategy used in PubMed** (*Date of search: 31 January 2026) | |
| #1 | "Stomach Neoplasms"[Mesh] |
| #2 | "Esophagogastric Junction"[Mesh] |
| #3 | (gastric cancer[Title/Abstract]) OR (stomach cancer[Title/Abstract]) OR (gastroesophageal junction cancer[Title/Abstract]) OR (gej cancer[Title/Abstract]) |
| #4 | #1 OR #2 OR #3 |
| #5 | advanced[Title/Abstract] |
| #6 | #4 AND #5 |
| #7 | "Pembrolizumab"[Mesh] |
| #8 | (Pembrolizumab[Title/Abstract]) OR (Keytruda[Title/Abstract]) OR (MK-3475[Title/Abstract]) |
| #9 | #7 OR #8 |
| #10 | "Receptor, ErbB-2"[MeSH] |
| #11 | ("HER2 positive"[Title/Abstract]) OR ("HER2-positive"[Title/Abstract]) OR ("HER-2 positive"[Title/Abstract]) OR ("HER-2-positive"[Title/Abstract]) OR ("HER2+"[Title/Abstract]) OR ("ERBB2 positive"[Title/Abstract]) OR ("ERBB2-positive"[Title/Abstract]) OR ("ErbB2 positive"[Title/Abstract]) OR ("ErbB2-positive"[Title/Abstract]) OR ("Human Epidermal Growth Factor Receptor 2 positive"[Title/Abstract]) OR ("Human Epidermal Growth Factor Receptor 2-positive"[Title/Abstract]) |
| #12 | #10 OR #11 |
| #13 | #6 AND #9 NOT #12 |
| Note: mh: MeSH terms; tiab: title/abstract; ti: title | |
| b. **Retrieval strategy used in EMBASE** (*Date of search: 31 January 2026) | |
| #1 | 'stomach cancer'/exp OR 'gastroesophageal junction cancer'/exp OR 'stomach cancer':ti,ab OR 'gastric cancer':ti,ab OR 'stomach neoplasms':ti,ab OR 'gastroesophageal junction cancer':ti,ab OR 'gej cancer':ti,ab |
| #2 | 'advanced':ti,ab |
| #3 | 'pembrolizumab'/exp OR 'pembrolizumab':ti,ab OR 'keytruda':ti,ab OR 'mk 3475':ti,ab |
| #4 | 'epidermal growth factor receptor 2'/exp OR 'her2 positive':ti,ab OR 'her2-positive':ti,ab OR 'her-2 positive':ti,ab OR 'her-2-positive':ti,ab OR 'her2+':ti,ab OR 'erbb2 positive':ti,ab OR 'erbb2-positive':ti,ab OR 'human epidermal growth factor receptor 2 positive':ti,ab OR 'human epidermal growth factor receptor 2-positive':ti,ab |
| #5 | #1 AND #2 AND #3 NOT #4 |
| c. **Search strategy used in Web of Science** (*Date of search: 31 January 2026) | |
| #1 | TTS=("stomach neoplasms" OR "stomach neoplasm" OR "stomach cancer*" OR "gastric cancer*" OR "stomach tumor*" OR "stomach tumour*" OR "stomach carcinoma*" OR "gastric tumor*" OR "gastric tumour*" OR "gastric carcinoma*" OR "esophagogastric junction" OR "gastroesophageal junction" OR "gastroesophageal junction cancer*" OR "gej cancer*") and Preprint Citation Index |
| #2 | TS=("advanced") and Preprint Citation Index |
| #3 | TS=("Pembrolizumab" OR "Keytruda" OR "MK-3475") and Preprint Citation Index |
| #4 | TS=("HER2 positive" OR "HER2-positive" OR "HER-2 positive" OR "HER-2-positive" OR "HER2+" OR "ERBB2 positive" OR "ERBB2-positive" OR "ErbB2 positive" OR "ErbB2-positive" OR "Human Epidermal Growth Factor Receptor 2 positive" OR "Human Epidermal Growth Factor Receptor 2-positive" OR "Receptor, ErbB-2") and Preprint Citation Index |
| #5 | ((#1 AND #2 AND #3) NOT #4) and Preprint Citation Index |
| d.**search strategy used in Scopus** (*Date of search: 31 January 2026) | |
| #1 | TITLE-ABS-KEY("stomach neoplasms" OR "stomach cancer*" OR "gastric cancer*" OR "esophagogastric junction" OR "gastroesophageal junction cancer*" OR "gej cancer*") |
| #2 | TITLE-ABS-KEY("advanced") |
| #3 | TITLE-ABS-KEY("pembrolizumab" OR "keytruda" OR "mk-3475") |
| #4 | TITLE-ABS-KEY("her2 positive" OR "her2-positive" OR "her-2 positive" OR "her-2-positive" OR "her2+" OR "erbb2 positive" OR "erbb2-positive" OR "human epidermal growth factor receptor 2 positive" OR "human epidermal growth factor receptor 2-positive" OR "receptor erbb-2") |
| #5 | #1 AND #2 AND #3 |
| #6 | #5 AND NOT #4 |
| e.**search strategy used in Ovid** (*Date of search: 31 January 2026) | |
| 1 | exp Stomach Neoplasms/ |
| 2 | exp Esophagogastric Junction/ |
| 3 | (gastric cancer* or stomach cancer* or gastroesophageal junction cancer* or gej cancer*).ti,ab. |
| 4 | 1 or 2 or 3 |
| 5 | advanced.ti,ab. |
| 6 | 4 and 5 |
| 7 | exp Pembrolizumab/ |
| 8 | (Pembrolizumab or Keytruda or MK-3475).ti,ab. |
| 9 | 7 or 8 |
| 10 | exp Receptor, ErbB-2/ |
| 11 | (HER2 positive or HER2-positive or HER-2 positive or HER-2-positive or HER2+ or ERBB2 positive or ERBB2-positive or ErbB2 positive or ErbB2-positive or Human Epidermal Growth Factor Receptor 2 positive or Human Epidermal Growth Factor Receptor 2-positive).ti,ab. |
| 12 | 10 or 11 |
| 13 | 6 and 9 |
| 14 | 13 not 12 |
| 15 | exp Radioactivity/ |
| f.**Search strategy used in Cochrane Library**(*Date of search: 31 January 2026) | |
| #1 | MeSH descriptor: [Stomach Neoplasms] explode all trees |
| #2 | MeSH descriptor: [Esophagogastric Junction] explode all trees |
| #3 | (("gastric cancer*") OR ("stomach cancer*") OR ("gastroesophageal junction cancer*") OR ("gej cancer*")):ti,ab,kw (Word variations have been searched) |
| #4 | #1 OR #2 OR #3 |
| #5 | (("advanced")):ti,ab,kw (Word variations have been searched) |
| #6 | #4 AND #5 |
| #7 | MeSH descriptor: [Pembrolizumab] explode all trees |
| #8 | (("Pembrolizumab") OR ("Keytruda") OR ("MK-3475")):ti,ab,kw (Word variations have been searched) |
| #9 | #7 OR #8 |
| #10 | MeSH descriptor: [Receptor, ErbB-2] explode all trees |
| #11 | (("HER2 positive") OR ("HER2-positive") OR ("HER-2 positive") OR ("HER-2-positive") OR ("HER2+") OR ("ERBB2 positive") OR ("ERBB2-positive") OR ("ErbB2 positive") OR ("ErbB2-positive") OR ("Human Epidermal Growth Factor Receptor 2 positive") OR ("Human Epidermal Growth Factor Receptor 2-positive")):ti,ab,kw (Word variations have been searched) |
| #12 | #10 OR #11 |
| #13 | #6 AND #9 NOT #12 |
| g.**Search strategy used in CINAHL**(*Date of search: 31 January 2026) | |
| S1 | (MH "Stomach Neoplasms+") OR (MH "Gastroesophageal Junction+") OR TI ( "gastric cancer*" OR "stomach cancer*" OR "gastroesophageal junction cancer*" OR "gej cancer*" ) OR AB ( "gastric cancer*" OR "stomach cancer*" OR "gastroesophageal junction cancer*" OR "gej cancer*" ) |
| S2 | TI ( advanced ) OR AB ( advanced ) |
| S3 | TI ( pembrolizumab OR keytruda OR "mk-3475" ) OR AB ( pembrolizumab OR keytruda OR "mk-3475" ) |
| S4 | TI ( "her2 positive" OR "her2-positive" OR "her-2 positive" OR "her-2-positive" OR "her2+" OR "erbb2 positive" OR "erbb2-positive" OR "human epidermal growth factor receptor 2 positive" OR "human epidermal growth factor receptor 2-positive" ) OR AB ( "her2 positive" OR "her2-positive" OR "her-2 positive" OR "her-2-positive" OR "her2+" OR "erbb2 positive" OR "erbb2-positive" OR "human epidermal growth factor receptor 2 positive" OR "human epidermal growth factor receptor 2-positive" ) |
| S5 | (MH "Receptor, ErbB-2") |
| S6 | S4 OR S5 |
| S7 | S1 AND S2 AND S3 |
| S8 | S7 NOT S6 |
| h.**Search strategy used in Clinicaltrials.gov**(*Date of search: 31 January 2026) | |
| #1 | Stomach Cancer OR Gastric Cancer OR Gastroesophageal Junction Cancer OR GEJ Cancer |
| #2 | advanced |
| #3 | #1 AND #2 |
| #4 | Pembrolizumab OR Keytruda OR "MK-3475" |
| #5 | #3 AND #4 |
| #6 | "HER2 positive" OR "HER2+" OR "ERBB2" |
| #7 | #5 NOT #6 |
